# Large-scale diet tracking data reveal disparate associations between food environment and diet

**DOI:** 10.1038/s41467-021-27522-y

**Published:** 2022-01-18

**Authors:** Tim Althoff, Hamed Nilforoshan, Jenna Hua, Jure Leskovec

**Affiliations:** 1 Allen School of Computer Science & Engineering, University of Washington, Seattle, WA USA; 2 Department of Computer Science, Stanford University, Stanford, CA USA; 3 Stanford Prevention Research Center, Department of Medicine, Stanford University School of Medicine, Stanford, CA USA; 4 Million Marker Wellness Inc., San Francisco, CA USA; 5 Chan Zuckerberg Biohub, San Francisco, CA USA

**Keywords:** Epidemiology, Risk factors

## Abstract

An unhealthy diet is a major risk factor for chronic diseases including cardiovascular disease, type 2 diabetes, and cancer^1–4^. Limited access to healthy food options may contribute to unhealthy diets^5,6^. Studying diets is challenging, typically restricted to small sample sizes, single locations, and non-uniform design across studies, and has led to mixed results on the impact of the food environment^7–23^. Here we leverage smartphones to track diet health, operationalized through the self-reported consumption of fresh fruits and vegetables, fast food and soda, as well as body-mass index status in a country-wide observational study of 1,164,926 U.S. participants (MyFitnessPal app users) and 2.3 billion food entries to study the independent contributions of fast food and grocery store access, income and education to diet health outcomes. This study constitutes the largest nationwide study examining the relationship between the food environment and diet to date. We find that higher access to grocery stores, lower access to fast food, higher income and college education are independently associated with higher consumption of fresh fruits and vegetables, lower consumption of fast food and soda, and lower likelihood of being affected by overweight and obesity. However, these associations vary significantly across zip codes with predominantly Black, Hispanic or white populations. For instance, high grocery store access has a significantly larger association with higher fruit and vegetable consumption in zip codes with predominantly Hispanic populations (7.4% difference) and Black populations (10.2% difference) in contrast to zip codes with predominantly white populations (1.7% difference). Policy targeted at improving food access, income and education may increase healthy eating, but intervention allocation may need to be optimized for specific subpopulations and locations.

## Abstract (web summary)

Studying diets is challenging, typically restricted to small sample sizes, single locations, and non-uniform design across studies. Here, the authors leverage food entry data of a popular diet tracking app to observe diet health and weight status, studying the associations of fast food and grocery access, income and education with diet health outcomes.

## Introduction

Dietary factors significantly contribute to risk of mortality and chronic diseases such as cardiovascular diseases, type 2 diabetes and cancer globally^1–3^. Emerging evidence suggests that the built and food environment, behavioral, and socioeconomic factors significantly affect diet^7^. Prior studies of the food environment and diet have led to mixed results^7–23^, and very few used nationally representative samples. These mixed results are potentially attributed to methodological limitations of small sample size, differences in geographic contexts, study population, and non-uniform measurements of both the food environment and diet across studies. Therefore, research with larger sample size and using improved and consistent methods and measurements is needed^9,24,25^.

**Fig. 1:**
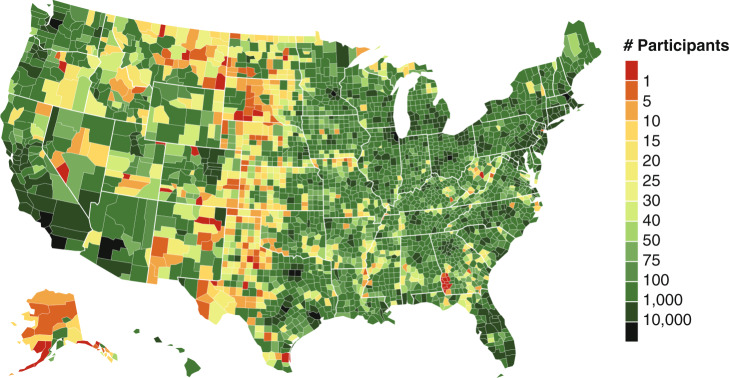
Number of participants in our study across U.S. counties. A choropleth showing the number of participants in each U.S. county. This country-wide observational study included 1,164,926 participants across 9822 U.S. zip codes that collectively logged 2.3 billion food entries for an average of 197 days each. This study constitutes the largest nationwide study examining the relationship between food environment and diet to date (e.g., with 511% more counties represented compared to BRFSS data^93^).

**Fig. 2:**
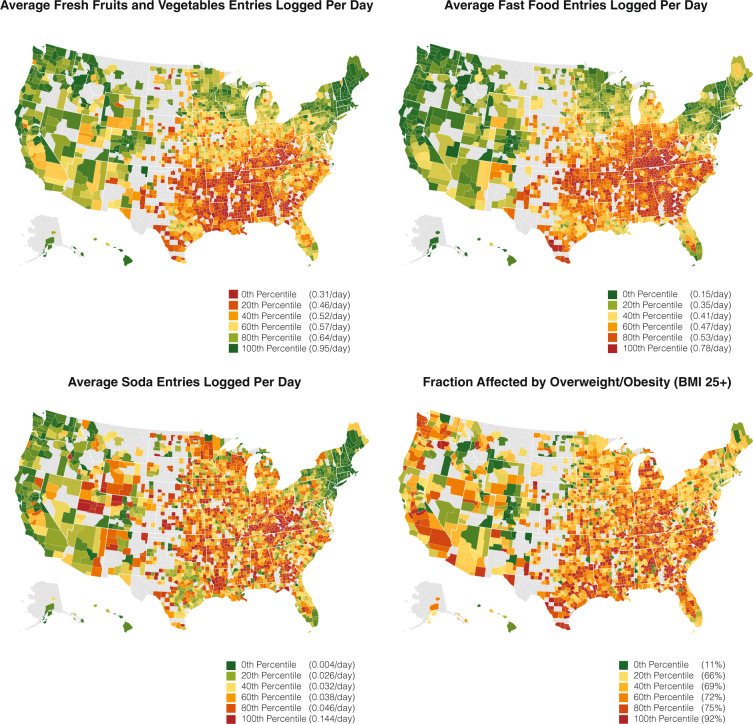
Dietary consumption and BMI status across U.S. counties. A set of choropleths showing the main study outcomes of the number of entries that are classified as fresh fruit and vegetables, fast food, and soda consumption as well as the fraction affected by overweight/obesity (BMI > 25) participants across the USA by counties with more than 30 participants. We observe that food consumption healthfulness varies significantly across counties in the United States.

Commercially available and widely used mobile applications allow the tracking of health behaviors and population health^26^, as recently demonstrated in physical activity^27,28^, sleep^29,30^, COVID-19 pandemic response^31–33^, women’s health^34^, as well as diet research^35–40^. With ever increasing smartphone ownership in the U.S.^41^ and the availability of immense geospatial data, there are now unprecedented opportunities to combine various data on individual diets, population characteristics (gender and ethnicity), socioeconomic status (income and educational attainment), as well as food environment at large scale. Interrogation of these rich data resources to examine geographical and other forms of heterogeneity in the effect of food environments on health could lead to the development and implementation of cost-effective interventions^42^. Here, we leverage large-scale smartphone-based food journals of 1,164,926 participants across 9822 U.S. zip codes (Fig. 1) and combine several Internet data sources to quantify the independent associations of food (grocery and fast food) access, income and educational attainment with food consumption and body-mass index (BMI) status (Fig. 2). This study constitutes the largest nationwide study examining the relationship between the food environment and diet to date.

## Results

### Data validation: diet tracking data correlates with existing large-scale measures

**Fig. 3:**
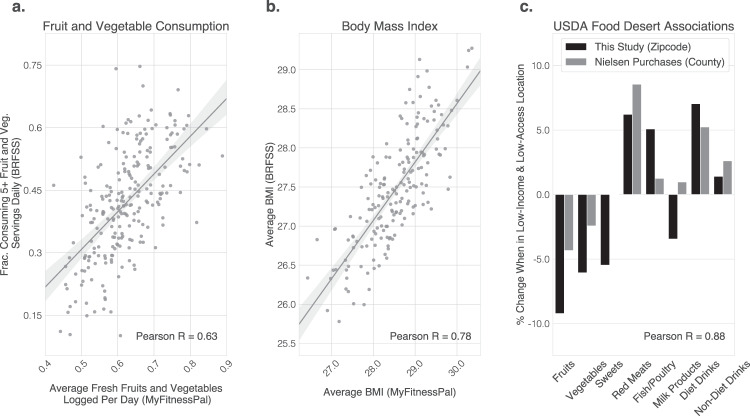
This studies’ smartphone-based food logs correlate with existing large-scale survey measures and purchase data. **a** Fraction of fresh fruits and vegetables logged is correlated with BRFSS survey data^43^ (Pearson Correlation *R* = 0.63, *p* < 10^−5^; Two-sided Student’s *t*-test; Methods). **b** Body-mass index of smartphone cohort is correlated with BRFSS survey data^44^ (Pearson Correlation *R* = 0.78, *p* < 10^−5^; Two-sided Student’s *t*-test; Methods). Lines in **a**, **b** show best linear fit along with shaded 95% bootstrap confidence intervals. **c** Digital food logs replicate previous findings of relative consumption differences in low-income, low-access food deserts based on Nielsen purchase data^45^ (Pearson Correlation *R* = 0.88, *p* < 0.01; Two-sided Student’s *t*-test; Methods). These results demonstrate that smartphone-based food logs are highly correlated with existing, gold-standard survey measures and purchase data.

To determine the ability of our dataset to identify relationships between fast food, grocery store access, income, educational attainment and diet health outcomes, we confirmed that this studies’ smartphone-based food logs correlate with existing large-scale survey measures and purchase data. Specifically, the fraction of fresh fruits and vegetables (F&V) that participants logged is correlated with Behavioral Risk Factor Surveillance System (BRFSS) survey data^43^ (Fig. 3a; Pearson Correlation *R* = 0.63, *p* < 10^−5^; Two-sided Student’s *t*-test; Methods). Further, the reported BMI of MyFitnessPal (MFP) participants is correlated with BRFSS survey data^44^ (Fig. 3b; Pearson Correlation *R* = 0.78, *p* < 10^−5^; Two-sided Student’s *t*-test; Methods). Lastly, the digital food logs data replicate previous findings of relative consumption differences in low-income, low-access food deserts based on Nielsen purchase data^45^ (Fig. 3c; Pearson Correlation *R* = 0.88, *p* < 0.01; Two-sided Student’s *t*-test; Methods). These results demonstrate that smartphone-based food logs are highly correlated with existing, gold-standard survey measures and purchase data.

### Associations between food environment, demographics and diet

**Fig. 4:**
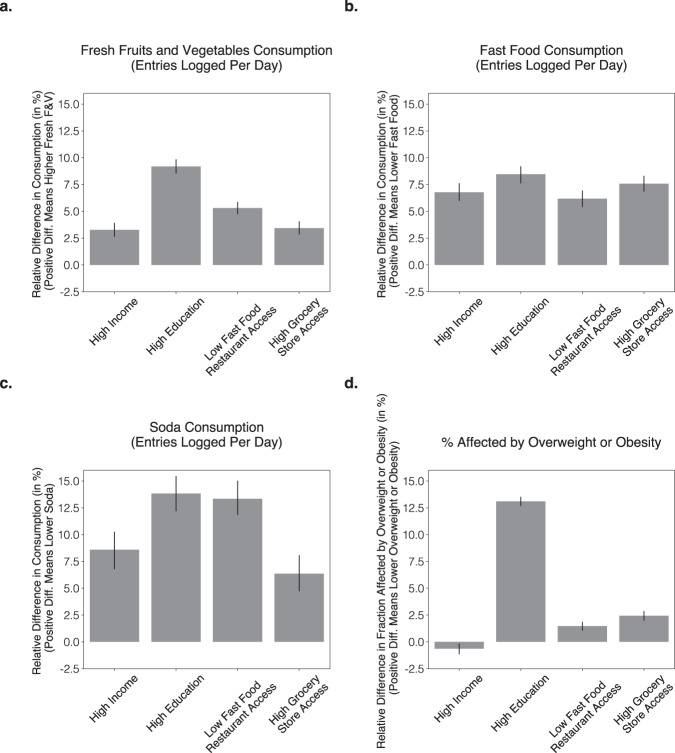
The association between income, educational attainment, grocery store and fast food access, with food consumption and BMI status. Independent contributions of high income (median family income higher than or equal to $70,241), high educational attainment (fraction of population with college education 29.8% or higher), high grocery store access (fraction of population that is closer than 0.5 miles from nearest grocery store is greater than or equal to than 20.3%), and low fast food access (less than or equal to 5.0% of all businesses are fast-food chains) on relative difference in consumption of **a** fresh fruits and vegetables, **b** fast food, **c** soda, and **d** relative difference in fraction affected by overweight or obesity (BMI > 25). Cut points correspond to median values. *Y*-axes are oriented such that consistently higher is better. Estimates are based on matching experiments controlling for all but one treatment variable, across *N* = 4911 matched pairs of zip codes (Methods). Bar height corresponds to mean values; error bars correspond to 95% bootstrap confidence intervals (Methods). While the most highly predictive factors vary across outcomes, only high educational attainment was associated with a sizeable difference of 13.1% in the fraction affected by overweight or obesity.

Using these data across all 9822 U.S. codes, we found that high income, high educational attainment, high grocery store access, and low fast food access were independently associated with higher consumption of fresh F&V, lower consumption of fast food and soda, and lower prevalence of BMI levels categorized as overweight or obesity (Fig. 4; BMI > 25). The only exception to this pattern was a very slight (0.6%) *positive difference* in BMI levels categorized as overweight or obesity associated with income. Specifically, in zip codes of above median grocery store access participants logged 3.4% more F&V, 7.6% less fast food, 6.4% less soda and were 2.4% less likely to be affected by overweight or obesity (all *P* < 0.001). In zip codes of below median fast food access participants logged 5.3% more F&V, 6.2% less fast food, 13.3% less soda and were 1.5% less likely to be affected by overweight or obesity (all *P* < 0.001). In zip codes of above median education, participants logged 9.2% more F&V, 8.5% less fast food, 13.8% less soda and were 13.1% less likely to be affected by overweight or obesity (all *P* < 0.001). Finally, in zip codes of above median household income (referred to as *higher income* below), participants logged 3.3% more F&V, 6.8% less fast food, 8.6% less soda (all *P* < 0.001), but had a 0.6% higher likelihood of being affected by overweight or obesity (*P* = 0.006). Note that the reported effect size are based on comparing above and below median zip codes for any given factor. We found a general pattern of consistent, and in many cases higher effect sizes when comparing top versus bottom quartiles (Supplementary Fig. 2), suggesting the possibility of a dose-response relationships across most considered variables. We found that zip codes with high educational attainment levels compared to low educational attainment levels had the largest relative positive differences across F&V, fast food, soda, and BMI levels categorized as overweight or obesity.

### Significant differences across zip codes with predominantly Black, Hispanic, and Non-Hispanic white populations

**Fig. 5:**
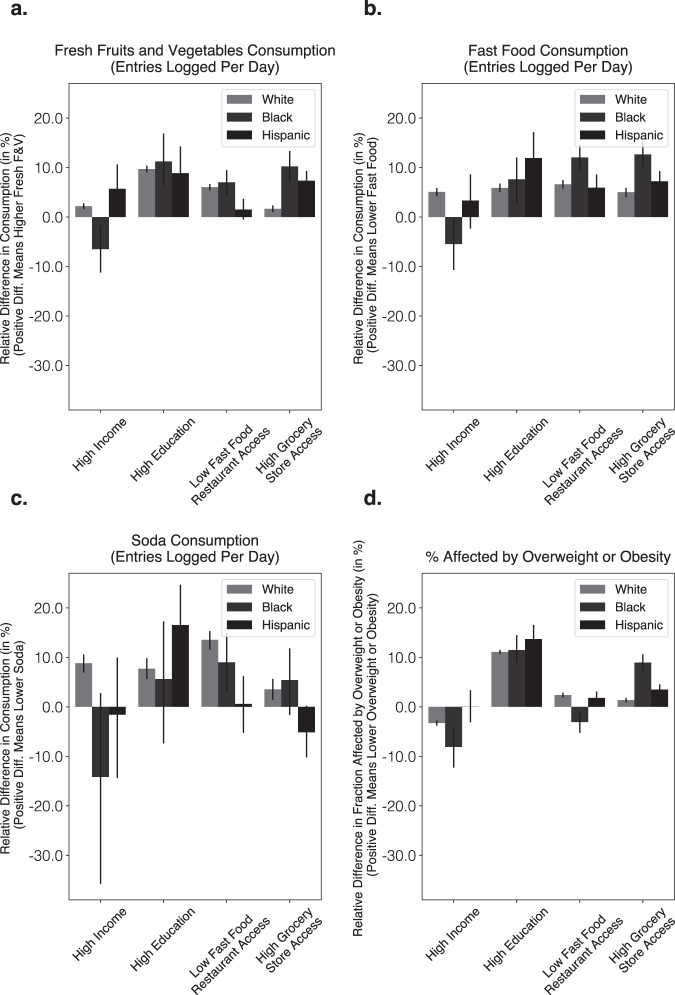
Effect sizes for food consumption and BMI status disaggregated across zip codes with predominantly Black, Hispanic, and non-Hispanic white populations (i.e., 50% or more). Independent contributions of high income (median family income higher than or equal to $70,241), high educational attainment (fraction of population with college education 29.8% or higher), high grocery store access (fraction of population that is closer than 0.5 miles from nearest grocery store is greater than or equal to than 20.3%), and low fast food access (less than or equal to 5.0% of all businesses are fast-food chains) on relative difference in consumption of **a** fresh fruits and vegetables, **b** fast food, **c** soda, and **d** relative difference in fraction affected by overweight or obesity (BMI > 25). Cut points correspond to median values. *Y*-axes are oriented such that consistently higher is better. Estimates are based on matching experiments controlling for all but one treatment variable, across *N* = 4277, 4102, 3510, 3205 matched pairs of non-Hispanic white-majority zip codes, treated on income, educational attainment, fast food access, grocery store access respectively; *N* = 42, 74, 259, 259 matched pairs of Black-majority zip codes, treated on income, educational attainment, fast food access, grocery store access respectively; *N* = 67, 61, 297, 471 matched pairs of Hispanic-majority zip codes, treated on income, educational attainment, fast food access, grocery store access respectively (Methods). Bar height corresponds to mean values; error bars correspond to 95% bootstrap confidence intervals (Methods). We observe significant differences in outcomes between zip codes with predominantly Black, Hispanic, and non-Hispanic white populations.

We separately repeated our data analyses within zip codes with predominantly Black (3.7%), Hispanic (5.6%), and non-Hispanic white populations (78.4%) (Fig. 5). Results within zipcodes with predominantly non-Hispanic white populations closely resembled results within the overall population, since most zip codes in this study had predominantly white populations (78.4%; not unlike the overall U.S. population at 61.3%)^46^. However, restricting our analyses to zip codes with predominantly Black and Hispanic populations led to significantly different findings. Specifically, within zip codes with predominantly Black populations we found associations of higher income in the inverse direction of the population average and towards low healthful food consumption, across four out of four outcome variables, resulting in lower F&V consumption (−6.5%), higher fast food consumption (5.5%), and higher likelihood of BMI levels categorized as overweight or obesity (8.1%). Higher income was also associated with higher soda consumption (14.1%) but was not statistically significant (*P* = 0.061). On the other hand, low fast food access and high educational attainment access were generally associated with higher diet health, with low fast food access correlating with the highest significant negative difference in fast food consumption (−12.0%) and high educational attainment with the highest positive difference in fresh fruit and vegetable consumption (11.2%), although lower fast food access was associated with worse outcomes for one of the outcome variables. Specifically, lower fast food access was associated with a slightly higher likelihood of being affected by overweight or obesity (3.1%). Higher grocery store access had a positive association with diet health across all outcome variables in zip codes with predominantly Black populations, and was associated with higher F&V consumption (10.2%), lower fast food consumption (12.6%), lower likelihood of BMI levels categorized as overweight or obesity (9.0%), and lower soda consumption (5.3%), although the association with soda consumption was not statistically significant (*P* = 0.060).

In contrast, within zip codes with predominantly Hispanic populations we found a significant association between higher, above-median, income and higher F&V consumption (5.7%), but not with the remaining three outcome variables. Zip codes with higher proportion of people with high educational attainment had the most positive association with diet health across all variables. Specifically, higher educational attainment was associated with higher F&V consumption (8.9%), lower fast food consumption (11.9%), lower soda consumption (16.5%), and lower likelihood of BMI levels categorized as overweight or obesity (13.7%). Higher grocery store access and lower fast food access had similar effect sizes as on the overall population for some outcome variables (i.e. similar associations with likelihood of BMI levels categorized as overweight or obesity and fast food consumption). However, in some cases the magnitude of association was higher (i.e. grocery store access was associated with 7.4% higher F&V consumption in areas with predominantly Hispanic population, which is more than twice than the difference within the overall population) and in others, unlike the overall population, there was no significant association (i.e. no significant relationship between fast food access on soda consumption, or between fast food access and F&V consumption).

### Summary

Few factors were consistently associated with better outcomes across all three subpopulations. Across all three groups, F&V consumption was significantly higher in zip codes with high grocery store access and high educational attainment. Fast food consumption was lower across all potential intervention targets besides higher income. Soda consumption was lowest most with lower fast food access for Black and white-majority zip codes, whereas it was lowest with higher educational attainment in Hispanic zip codes. Lastly, BMI levels categorized as overweight or obesity were far lower with higher educational attainment levels compared to all other intervention targets, across all three groups.

## Discussion

Commercially available and widely used mobile applications and devices enable the individuals to track their own health, and in aggregate may inform our understanding of population health. These emerging data sources capture health behaviors from millions of participants^26^ and have uniquely enabled large-scale research studies, including in physical activity^27,28^, sleep^29,30^, COVID-19 pandemic response^31–33^, women’s health^34^, as well as diet research^35–40^.

While many of our results were consistent with previous studies^47–49^, importantly, we found that zip codes with higher proportion of people with high educational attainment had the largest relative difference in the likelihood of BMI levels categorized as overweight or obesity (13.1% lower). It is well established that social determinants of health are linked to obesity^50–52^. As an important component of social determinants of health, our study suggests that having higher educational attainment is the most predictive of reduced overweight and obesity for all ethnicities.

When we restricted our analyses to zip codes with predominantly Black, Hispanic, and non-Hispanic white populations, we found the independent associations of food access, income and educational attainment with food consumption and BMI status varied significantly across these three groups. These findings suggest that tailored intervention strategies are needed based on neighborhood population distributions, assets and contexts.

Within zip codes with predominantly Black populations, the association between having higher income and diet health was negative. Having higher income was associated with lower F&V consumption, higher fast food and soda consumption, and higher likelihood of overweight and obesity. This could be explained by the “diminishing return hypothesis”, which suggests that Black people receive fewer protective health benefits from increases in SES than white people^53,54^. A combination of factors, including neighborhood economic disadvantage^55,56^, racial/ethnic discrimination^57,58^, and stress associated with educational attainment and mobility^59^, may prevent Black people from higher SES backgrounds from achieving their fullest health potential relative to white people^60^.

Within zip codes with predominantly Hispanic populations, higher income was not associated with lower likelihood of BMI levels categorized as overweight or obesity. The absence of a relationship between higher income and BMI, compared to in zip codes with predominantly Black and non-Hispanic white populations, could be partially explained by the “Hispanic health paradox” and “Hispanic health advantage”^61–66^. The Hispanic health paradox suggests that even though the first-generation Hispanic people have lower SES, they experience better health outcomes including lower prevalence of cardiovascular diseases, asthma, diabetes and cancer compared to those who were U.S.-born^61–63^. Hispanic health advantage suggests that Hispanic people have lower rates of harmful health behaviors, such as smoking, which in turn positively influence other health outcomes compared to non-Hispanic white people^61,64–66^. Additionally, through acculturation or adopting American culture, Hispanic immigrants may engage in less healthy behaviors, which in turn put themselves at higher risk for chronic diseases^61–63,67–71^.

While it is challenging to close the education and income gaps, establishing more grocery stores and limiting fast food restaurant access may help improve diet health across the population. Previous reviews suggested that government policies that addressing food affordability and purchase, such as the Healthy Food Financing Initiative (HFFI), increasing food stamp (SNAP) benefit and provide incentives to create healthy retail food environment have been effective in reducing food insecurity and dietary behaviors^72–77^. While several studies showed that the establishments of new supermarkets had little improvement in BMI^78–80^; however, the investments in the new supermarkets have improved economic opportunity and social cohesion^81–83^. Our results showed that higher grocery store access was associated with 2–3 times higher fresh fruit and vegetable consumption and lower fast food consumption more for Black people than for white people. Although previous literature has shown null effects of grocery store access^84,85^, these studies have focused on the general population, which is white-skewed. Therefore, policies and strategies in increasing grocery store access and decreasing fast food access could potentially be the most effective approaches in changing dietary habits among locations with predominantly Black populations.

Furthermore, having more grocery store access and lower fast food access, in the food environment may work in synergistic ways that may lead to even lower obesity prevalence and obesity-related lifestyle and behavior changes. This is demonstrated in a recent study by Cantor et al. that HFFI boosted the effects of SNAP participation on improving food security and healthy food choices in food desserts^86^. This synergy could be multiplied when combining with effective education programs that could potentially lower obesity prevalence further by increasing individuals’ SES (e.g., income and educational attainment)^87,88^, health literacy and behaviors^87–91^, as well as sense of control and empowerment^92^.

Due to the cross-sectional nature of the study, we were not able to make any causal inferences between SES, food environment variables, dietary behavior, and BMI, as unobserved neighborhood and individual demographic and social characteristics could lead to confounding. However, we used a matching-based approach to mimic a quasi-experimental design to disentangle the individual associations of income, educational attainment and food access with participants’ food consumption. Our analysis did not include other demographic variables such as gender and age, as both variables were naturally balanced across treatment and control groups and we observed minimal zipcode-level correlations between age/gender and any of our four outcome measures (Supplementary Table 13). In addition, we confirmed that results were virtually identical (Pearson Correlation *R* = 0.95), when explicitly controlling for age and gender in our matching-based approach. However, we jointly considered the potential impacts of neighborhood income, neighborhood educational attainment and food environment access on participants’ food consumption with consistent measures across the U.S., whereas previously published studies examined one or a few at a time. Our study population, based on a sample of MFP users, is an imperfect representation of the United States national population. Comparing our study population to nationally representative survey data, we found that our study population had significant overlap with the U.S. national population in terms of population demographics, educational attainment and BMI status, but that it was skewed towards women and higher income (Supplementary Table 3). We used individuals’ food loggings to estimate their consumption (specifically, the number of food entries as the logged amount consumed varied highly across foods without standardization; e.g., specifying weight, volume, or number). Food loggings may not capture what individuals actually ate and participants may be particularly motivated or care about their diet and weight. Importantly, we conducted multiple validation experiments through comparisons with high quality and highly representative datasets which demonstrated high correlations to gold-standard approaches (Fig. 3). The majority of food environment studies used screeners, food frequency questionnaires or 24-h recalls for dietary assessment, and very few used diaries^9^. In contrast, our participants logged their food intakes for an average of 197 days each. We also harnessed other large datasets such as Yelp to examine participants’ food environments. Considering both the strengths and limitations of this study, more research is needed especially based on longitudinal study design and detailed individual level data to enable causal inference and precise interpretation of the results.

In conclusion, we analyzed 2.3 billion food intake logs and BMIs from 1.2 million MFP smartphone app participants over 7 years across 9822 zip codes in relation to educational attainment, ethnicity, income, and food environment access. Our analyses indicated that higher access to grocery stores, lower access to fast food, higher income and educational attainment were independently associated with higher consumption of fresh F&V, lower consumption of fast food and soda, and lower likelihood of being affected by overweight or obesity, but that these associations varied significantly across zip codes with predominantly Black, Hispanic and white subpopulations. Policy targeted at improving food access, income and education may increase healthy eating. However, intervention allocation may need to be optimized for specific subpopulations and locations.

## Methods

### Study design and population

We conducted a United States countrywide cross-sectional study of participants’ self-reported food intake and BMI in relation to zip code level demographic (educational attainment, ethnicity), socioeconomic (income), and food environment factors (grocery store and fast food access) by combining datasets from MFP, US Census, USDA and Yelp.

Overall, this cross-sectional matching-based study analyzed 2.3 billion food intake logs from U.S. smartphone participants over 7 years across 9822 zip codes, which is 24% of overall USA zip codes (U.S. has a total of 41,692 zip codes). Participants were users of the MFP app, a free application for tracking caloric intake. We analyzed anonymized, retrospective data collected during a 7-year observation period between 2010 and 2016 that were aggregated to the zip code level. Comparing our study population to nationally representative survey data, we found that our study population had significant overlap with the U.S. national population in terms of population demographics, educational attainment and BMI status, but that it was skewed towards women and higher income (Supplementary Table 3). Our matching-based statistical methodology controls for observed biases between comparison groups in terms of income, educational attainment, grocery store access, and fast food access (Methods: Statistical Analysis). Data handling and analysis was conducted in accordance with MFP policies and with the guidelines of the Stanford University Institutional Review Board.

### Study data: MyFitnessPal

We compute outcome measures of food consumption and BMI status from 2.3 billion food intake logs by a sample of 1,164,926 U.S. participants of the MFP smartphone application to quantify food consumption across 9822 zip codes. The scale and geographic distribution of our study participants, as well as our outcome measures, are illustrated in Figs. 1 and 2 respectively. To ensure participant privacy as well as reliability of our measures, we decided to only include zip codes in which we had access to 30 or more participant food logs, which reduced the dataset size from 27,027 zip codes (spanning 3117 counties) to the final 9822 zip codes (spanning 1730, or 55% of all counties in the United States). Nevertheless, the geographical breadth of this dataset far exceeds existing food surveys. For example, our final dataset contained 511% more counties than the BRFSS survey of 283 counties, with 370% more participants per county on average^93^. While size and coverage compare favorably to BRFSS, it is important to understand what is not covered by our study. Figure 1 illustrates that our study lacks representation in the Midwest of the USA as well as in Alaska. In our study data, we further observed under-representation of zip codes with majority non-white population (Supplementary Table 3) and rural zip codes (RUCA codes 7 through 10^94,95^), as well as over-representation of high-income zip codes (median family income higher than $70,241).

During the observation period from January 1, 2010 to November 15, 2016, the average participant logged 9.30 entries into their digital food journal per day. The average participant used the app for 197 days. All participants in this sample used the app for at least 10 days. We classified the 2.3 billion food intake entries into three categories of public health interest, fresh F&V, fast food, and sugary non-diet soda, and excluded them from analysis if they did not match these categories. Our classification method is consistent with USDA MyPlate with one divergence of the exclusion of juices. The healthiness of juice as a fruit and vegetable serving is contested due to its sugar content and limited nutritional profile^96–98^. For more details on the definition of a food entry, our classification method, and the choice of outcome measure, see Details on outcome measures subsection in Methods.

We intentionally use a cross-sectional rather than longitudinal study design, since fine-grained and large-scale temporal data on changes in the food environment were not available.

### Study data: demographic and socioeconomic factors

We obtained data on demographic and socioeconomic factors from CensusReporter^99^. Specifically, for each zip code in our data set we obtained median family income, fraction of population with college education (Bachelor’s degree or higher), and fraction of population that is white (not including Hispanic), Black, or Hispanic from the 2010 to 2014 American Community Survey’s census tract estimates^99^. While data were available only on zip code level, previous studies have shown that area-level income measures are meaningful for health outcomes and describe unique socioeconomic inequities^100^.

### Study data: grocery store and fast food access

Grocery store access was defined as the fraction of population that is more than 0.5 miles away from a grocery store following the food desert status definitions from the USDA Food Access Research Atlas^101^. Contrary to the USDA definition, we found evidence that even in rural zipcodes, the fraction of population greater than 0.5 miles away from grocery stores has the strongest association with food consumption (compared to 10 and 20 miles away), and thus we used 0.5 miles as the threshold across rural and urban zipcodes (Methods: Details on food environment measures). We measured fast food access through the fraction of restaurants that are fast food restaurants within a sample from Yelp, querying the nearest 1000 businesses from the zip code’s center, up to a maximum radius of 40 km (25 miles). See subsections Data Validation and reproducing State-of-the-art Measures using Population-scale Digital Food Logs for details and validation of these objective food environment measures.

We release all data aggregated at zipcode level in order to enable validation, follow-up research, and use by policy makers.

### Details on food environment measures

We obtained data on grocery store access (fraction of population that is more than 0.5 miles away from grocery store) and food desert status from the USDA Food Access Research Atlas^101^. A census tract is considered a food desert by the USDA if it is both low-income (defined by Department of Treasury’s New Markets Tax Credit program) and low-access, meaning at least 500 people or 30 percent of residents live more than 0.5 miles from a supermarket in urban areas (10 miles in rural areas)^45^.

Although the USDA uses different thresholds for urban and rural areas (0.5 and 10 miles respectively), we found that even in rural zipcodes (defined as USDA rural-urban continuum RUCA scores of 7 through 10^94,95^), the fraction of population that is farther than 0.5 miles from grocery stores had the highest correlation to fruit and vegetable consumption (Pearson Correlation *R* = −0.20), compared to 1 miles (Pearson Correlation *R* = −0.17), 10 miles (*R* = −0.05), and 20 miles (Pearson Correlation *R* = 0.03). This suggests that the fraction of the population farther than 0.5 miles from a grocery store has the strongest relationship with healthy food consumption, *even in rural zipcodes*. Hence, we decided used 0.5 miles distance as a standard measure of grocery store access for rural and urban zip codes, contrary to the USDA definition. We subsequently sanity checked for any downstream confounding of urbanicity in our primary matching experiment of above/below median grocery store access, and found a negligible difference (Standardized Mean Difference (SMD) of 0.18) in urbanicity between control and treatment, suggesting that the effect size was not due to grocery store distance functioning as a proxy for urbanicity, but rather directly due to differential grocery store access.

We aggregated these data from a census tract level to a zip code level using USPS Crosswalk data, which provides a list of all census tracts which overlap with a single zip code^102^. We related these data on census tract level to the zip code level by taking the weighted average of each census tract food environment measure (both grocery store access and food desert status), weighted by the number of people in the tract^102^. For instance, if zip code A overlapped with Census Tract A (2500 people, food desert) and Census Tract B (7500 people, not a food desert), the food desert measure of zip code A would be estimated as 25%. We defined the binary threshold for food desert, used in Fig. 3, as 50% or higher.

We measured fast food access through the fraction of restaurants in a zip-code that are fast food restaurants. Data on local restaurants and businesses were obtained through the Yelp API^103^. For each zip code, we consider up to 1000 restaurant businesses that are nearest to the zip code center up to a distance of 40 km (67.8% of zip code queries resulted in 1000 restaurant businesses within 40 km; Yelp API results are restricted to 1000 results). This resulted in a varying sample radius depending on urbanicity. For example, Urban zipcodes (RUCA code of 1) had an average effective centroid size of 15 miles, which we calculated by taking the distance from the zipcode center to the furthest restaurant returned by Yelp. We further used Yelp-based environment variables that we expected *not* to influence food consumption, such as the availability of waterproofing services, countertop installers, or electronic stores, as null experiments to demonstrate discriminant validity of our statistical analysis pipeline (see Supplementary Fig. 3).

### Details on outcome measures (food consumption and BMI status)

We used 2.3 billion food intake logs by a sample of 1,164,926 U.S. participants of the MFP smartphone application to quantify food consumption across 9822 zip codes. During the observation period from January 1, 2010 to November 15, 2016, the average participant logged 9.30 entries into their digital food journal per day. The average participant used the app for 197 days. All participants in this sample used the app for at least 10 days.

Clustering of food consumption observations within individuals and zip codes was handled through multiple levels of aggregation. First we aggregated within participant and day (i.e., someone eating a banana at breakfast and another for dinner), then we aggregated across all days with tracking within each participant, and then across all participants within one zip code. We computed non-parametric confidence intervals and *p*-values through bootstrapping with 1000 replications on zip code level (last level of aggregation)^104^.

The unit of analysis for each zipcode was the average number of daily entries per person. An entry is a single food consumption event logged in the app MFP. Each entry contains a separate food component (e.g., banana, yogurt, hamburger, ...), brand name (e.g., “Campbells”), description (e.g., “Chicken Soup”), serving size unit (e.g., “cup”), and number of servings (e.g., “1”). Supplementary Fig. 1 shows the application interface for logging a food entry (e.g., 1 Banana from Whole Foods). We decided to use entries based on the observation that there was little variance in the number of servings per food category logged by participants in a single entry, and since the amount consumed varied highly across foods without standardization (e.g., specifying weight, volume, or number). Participants typically log “standard portion sizes” of each food individually (e.g., one bowl of cereal, one banana) on the MFP app. For example, for participants that logged a banana, and listed the serving size as “Banana”, the median entry was for 1 banana, the mean was for 0.88 bananas, and 95% of food entries were for between 0.5 to 1.5 bananas. The MFP app strongly encourages this behavior through a large library of foods to log that follows these standard portion sizes.

We classified all entries into three categories using brand name and description, and three separate binary classifiers: fresh F&V (through a proprietary classifier by MFP which used key words in the brand name and description), fast food (if the brand name contained the name of a fast food chain listed in Supplementary Table 8, and sugary (non-diet) soda (if the brand name contained the name of a soda drink listed in Supplementary Table 9 and the description did not contain “diet”, “lite”, “light”, or “zero”). In all cases, descriptions, as well fast food and soda drink keywords, were normalized by lower-casing and removing punctuation. Each binary classifier thus took a food entry as input (i.e., “Coca Cola, Diet Cherry Coke, 8oz”) and outputted a binary label (i.e., soda or NOT soda). Entries which were predicted to be in none of the three categories based on all three models were excluded from the study.

Our classification method for fresh F&V is consistent with USDA MyPlate. The only divergence from USDA MyPlate is that we intentionally excluded juices, for which MFP has a separate classifier, which does not separate sweetened juice drinks or sports drinks and 100% juice. For our definition of Fresh F&V, we chose to exclude juices because the healthiness of juice as a fruit and vegetable serving is contested^96,98^, as even 100% fruit juices are typically high in sugar and calories, and low in fiber, and vegetable juices are often mixed with other high-sugar ingredients. We thus took a conservative approach to estimating diet healthiness by excluding these food entries.

We evaluated the accuracy of each of the three binary classification model by estimating the precision (# True Positive / # Predicted Positive) from a random sample of 50 entries belonging to each category. Precision estimates are summarized in Supplementary Table 1, and Supplementary Tables 10, 11, and 12 show random samples of 50 food items from all elements predicted to be in each category (where asterisk “*” indicates an incorrect prediction). Note that across 2.3 billion food logs it was not possible to measure recall, but were able to measure precision by manually inspecting the food entry brand and description and assigning it a category.

We then calculated the average number of food entries logged per participant, per day, for each of the F&V, fast food, and soda categories (e.g. average number of F&V logged per participant per day), excluding days in which the participant was inactive (i.e., consistently did not log anything). Finally, we aggregated these participant-level measures to the zip code level by taking the mean of each category’s measure for all participants in each zip code. We further used BMI health in each zip code as a BMI status outcome, specifically the fraction of participants in a zip code which are affected by overweight or obesity (BMI > 25). BMI was self-reported by participants of the smartphone application (99.92% of participants did report BMI). Supplementary Table 2 shows basic summary statistics for the outcome measures used in this study. In our statistical analyses, we compared two sets of zip codes that differ in a dimension of interest (e.g., grocery store access access) as treatment and control group and use the relative difference in F&V consumption, fast food consumption, soda consumption, and BMI health of the treated group relative to the control group. To generate confidence intervals, as well as to compute *p*-values to test for statistical significance of differences in outcome, we use non-parametric bootstrap resampling with 1000 replications^104^. Specifically, we follow the method proposed by Austin and Small^105^, which is to draw bootstrap samples post-matching from the matched pairs in the propensity-score-matched sample after the Genetic Matching stage^106^. We confirmed the validity of this method empirically by also calculating *t*-tests for each experiment, which gave qualitatively similar results. We note that we perform bootstrapping on zip code level (highest level of aggregation). While, multilevel bootstrapping methods exist, they do not scale well with our dataset size of 2.3 billion food items. However, due to the large number of 9822 zip codes our analyses are well-powered statistically even with bootstrapping at zip code level.

### Data validation

We find that our study population has significant overlap with the U.S. national population (Supplementary Table 3) but is skewed towards women and higher income. We demonstrate that food consumption measured based on this population are highly correlated with state-of-the-art measures (Fig. 3). Smartphone apps such as MFP feature large databases with nutritional information and can be used to track one’s diet over time. Previous studies have compared app-reported diet measures to traditional measures including 24-h dietary recalls and food composition tables. These studies found that both measures tend to be highly correlated^107,108^, but that app-reported measures tend to underestimate certain macro- and micronutrients^107,108^, especially in populations that were previously unfamiliar with the smartphone applications^109^. In contrast, this study leverages a sample of existing participants of the smartphone app MFP. Yelp data has been used in measures of food environment^110^ and a study in Detroit found Yelp data to be more accurate than commercially available databases such as Reference USA^111^. This study uses a combination of MFP data to capture food consumption, Yelp, and USDA data to capture food environment, and Census data to capture basic demographics. As a preliminary, basic test, we investigated correlations between the Mexican food consumption, the fraction of Mexican restaurants, and the fraction of Hispanic people in the population, on a zip code level. We found that Mexican food consumption (entries labeled as Mexican food by a proprietary MFP classifier, logged per participant, per day) was correlated with the fraction of Mexican restaurants (Pearson Correlation *R* = 0.72; < 10^−4^) and the fraction of Hispanic people in the population (Pearson Correlation *R* = 0.54; *P* < 10^−4^). Further, the fraction of Mexican restaurants was correlated with the fraction of Hispanic people in the population as well (Pearson Correlation *R* = 0.51; *P* < 10^−4^).

### Reproducing state-of-the-art measures using population-scale digital food logs

A primary concern in studying diet health via food logs is the bias inherent to the MFP population, which is not a representative sample of the US population. To investigate the applicability of population-scale digital food logs to study the relationship between food environment, income and educational attainment with food consumption, we measured the correlation between our smartphone app-based measures and state-of-the-art measures of food consumption including the BRFSS, based on representative surveys of over 350,000 adults in the United States^43,44^, and the Nielsen Homescan data^112^, which is a nationally representative panel survey of the grocery purchases of 169,000 unique households across the United States, based on UPC records of all consumer packaged goods participants purchased from any outlet (Fig. 3). We used the latest survey data from BRFSS^43,44^ available at the county-level. Specifically, we used variables FV5SRV from BRFSS 2011 representing the faction of people eating five or more servings of fresh fruit and vegetables^43^, and BMI5 from BRFSS 2012 representing BMI^44^. We compare against BRFSS rather than National Health and Nutrition Examination Survey (NHANES), since BRFSS is significantly larger than NHANES, it is remotely administered matching our study, and it has much better geographical coverage than NHANES and geographical comparisons are central to our study. Despite these advantages, no reference dataset is without limitations^113–115^, motivating this study’s use of large-scale digital food journals.

Comparing our data to BRFSS on county level, we found moderate to high correlations between the amount of fresh F&V consumed (Fig. 3a, Pearson Correlation *R* = 0.63, *p* < 10^−5^) and BMI (Fig. 3b, Pearson Correlation *R* = 0.78, *p* < 10^−5^). We further compared to published results by the USDA^45^, which used data from the 2010 Nielsen Homescan Panel Survey that captured household food purchases for in-home consumption (but did not capture restaurants and fast food purchases). We attempted to reproduce published findings on the differences in low-income, low-access communities (food deserts) compared to non-low-income, non-low-access communities^45^ across categories of fruit, vegetable, sweets, red meat, fish/poultry, milk products, diet drinks, and non-diet drinks (Table 4 in Rahkovsky and Snyder^45^). We used proprietary MFP classifiers to categorize foods logged into these categories. We found that our app-based food logs were very highly correlated with previously published results (Fig. 3c, *R* = 0.88, *p* < 0.01) and that the absolute differences between food deserts and non-food deserts were stronger in the MFP data compared to Nielsen purchase data. Overall, these results demonstrate convergent validity and suggest that the employed non-representative sample of population-scale digital food logs can reproduce the basic dynamics of traditional, state-of-the-art measures, and they can do so at massive scale and comparatively low cost.

### Statistical analysis

In this large-scale observational study, we used a matching-based approach^116,117^ to disentangle contributions of income, educational attainment, grocery store access, and fast food access on food consumption. We considered multiple statistical strategies, including regression modeling and propensity score matching. We decided to employ a full matching on all variables, which avoids parametric assumptions and is a more conservative method for matching than for example propensity score-based techniques^117^. To estimate the treatment effects of each of these factors, we divided all available zip codes into treatment and control groups based on a median split; that is, we estimated the difference in outcomes between matched above-median and below-median zip codes. We created matched pairs of zip codes by selecting a zip code in the control group that is closely matched (i.e., less than 0.25 SMD between the treated and control groups)^117^ to the zip code in the treatment group across all factors, except the treatment factor of interest. Since we repeated this matching process for each zip code in the treatment group, this approach estimated the Average Treatment Effect on the Treated (ATT). Through this process, we attempted to eliminate variation of plausible influences and to isolate the effect of interest. We repeated this process for each treatment of interest; for example for the results presented in Fig. 4, we performed four matchings, one for each of income, educational attainment, grocery store access and fast food access. For the sub-population experiments (Fig. 5), we repeated the same method on the subset of the zip codes in which the majority of inhabitants were of a particular ethnic group. Lastly, although we considered controlling for age and gender in the matching procedure, as these are related to diet health at the individual-level, we did not include them in our final analysis after observing (1) minimal zipcode-level correlations between age/gender and any of our four outcome measures (Supplementary Table 13; largest Pearson Correlation was 0.12) and (2) virtually identical results (Pearson Correlation *R* = 0.95) when comparing before and after controlling for age and gender by adding them the genetic matching algorithm. See subsection on Details on Matching Approach for further details and statistics that demonstrate that treatment and control groups were well-balanced on observed covariates after matching.

We tested discriminant validity of our statistical approach by measuring the effect of null-treatments that should not have any impact on food consumption. We chose examples of null-treatments by selecting variables that had little correlation with study independent variables (income, educational attainment, grocery store access, fast food access) and were plausibly unrelated to food consumption. This selection process lead to use of the fraction of countertop installers, electronics stores, and waterproofing services nearby as measured through Yelp. Applying our analysis pipeline to these null-treatments, we found that all of these null-treatments had zero effect on food consumption. This demonstrated that our statistical analysis approach did not produce measurements that it was not supposed to measure; that is, discriminant validity (Supplementary Fig. 3 and Supplementary Table 7).

### Details on matching approach

Specifically, we use a one-to-one Genetic Matching approach,^106^ with replacement, and use the mean of the SMD between treatment and control groups, across all matched variables, as the Genetic Matching balance metric in order to maximize balance (overlap) between the treated and the control units. Some definitions of SMD use the standard deviation in the overall population before matching^116^. However, we choose the standard deviation in the control group post-matching, which typically is much smaller and therefore gives more conservative estimates of balance between treated and control units^118^.

After matching, we evaluated the quality of balance between the treated and the control units by the SMD across each of the variables that were controlled for and included in the matching process. A good balance between treated and control groups was defined as a SMD of less than 0.25 standard deviations^117^ across each variable. By default, we do not enforce a caliper in order to minimize bias in matching process, although in rare cases in which a good balance was not achieved, a caliper was enforced, starting at 2.5 standard deviations between matched and controlled units, and decreased by 0.1 until the matched and control groups had a SMD smaller than 0.25 across all matched variables.

For the vast majority of matching experiments the SMD across all matched variables was well below 0.25, with a mean of 0.040 and median of 0.016 for the four overall population matching experiments. The SMD for the ethnicity-majority zipcode experiments was slightly higher, but still very significantly below 0.25 across all 12 experiments, with a mean of 0.055 and median of 0.036. Thus, no caliper was necessary to ensure a good balance, with the exception of one out of the 12 of sub-population experiments (white, high educational attainment). Detailed balancing statistics for each of the matches are available in the Supplementary Information (Supplementary Tables 14–36b), as well as a supplementary matching experiment in which a top/bottom quartile split was used instead of a median split (Supplementary Fig. 2).

### Details on the use of zip codes

A zip code is a postal code used by the US Postal Services. Zip codes consist of 5 digits and were introduced in their current form in 1983 in order to provide granular demarcations of US geography for mail purposes^119^. Most previous surveys such as BRFSS aggregate individuals at the less fine-grained levels of granularity: city, county, or MSA (Metropolitan statistical area) level. By contrast, we chose to use zip codes in order to study diet health and obesity at a more fine-grained level of analysis. As a point of reference, there are currently 41,692 zip codes in the USA compared to 3143 counties and county equivalents (i.e., 13.2 zip codes per county on average). Zip codes are on average 91 square miles and contain 7872 people^120^, compared to counties and county-equivalents which are on average 1208 square miles and contain 104,422 people^121^. Neighboring zip codes which may be in the same county have sharply contrasting demographics^122^. A zip code-level analysis better enables us to measure the disparate impacts of educational attainment, income, and food environment on diet health and obesity, and to stratify our analyses by ethnicity.

### Disclaimer

The content is solely the responsibility of the authors and does not necessarily represent the official views of the NIH or sponsors.

### Reporting summary

Further information on research design is available in the Nature Research Reporting Summary linked to this article.

## Supplementary information

Supplementary Information

Reporting Summary

## Data Availability

Data are available at http://snap.stanford.edu/dietdisparities.

## Code availability

Code is available at http://snap.stanford.edu/dietdisparities.
