# Supplementary material for: Large-scale diet tracking data reveal disparate associations between food environment and diet
Source: Nat Commun. 2022 Jan 18;13:267. doi: 10.1038/s41467-021-27522-y (PMC8766578; doi:10.1038/s41467-021-27522-y)
Supplement: Supplementary file 1 — Supplementary Information [file 41467_2021_27522_MOESM1_ESM.pdf]

Large-scale Diet Tracking Data Reveal Disparate Associations between Food Environment and Diet

Tim Althoff,[1*] Hamed Nilforoshan[2], Jenna Hua[3,4], Jure Leskovec[2,5]

[1]Allen School of Computer Science & Engineering, University of Washington

[2]Department of Computer Science, Stanford University

[3]Stanford Prevention Research Center, Department of Medicine, Stanford University School of Medicine

[4]Million Marker Wellness Inc.

[5]Chan Zuckerberg Biohub, San Francisco, CA

[*]To whom correspondence should be addressed;

E-mail: althoff@cs.washington.edu

Supplementary Information

- Supplementary Figures
- Supplementary Tables

List of Figures

1 **MyFitnessPal food logging app interface.** A screenshot of the MyFitnessPal app, showing the information collected for each food entry. 7

2 Matching experiments using quartile instead of median split. Note the consistent and in many cases larger effect sizes compared to Figure 4. Estimates are based on matching experiments controlling for all but one treatment variable, across $N = 2456$ matched pairs of zip codes (Methods). Bar height corresponds to mean values; error bars correspond to 95% bootstrap confidence intervals (Methods). . . 13

3 **Demonstration of discriminant validity of statistical approach.** We measured the effect of null-treatments that should not have any impact on food consumption. We chose examples of null-treatments by selecting variables that had little correlation with study independent variables (income, educational attainment, grocery store access, fast food access) and were plausibly unrelated to food consumption. This selection process lead to use of the fraction of countertop installers, electronics stores, and waterproofing services nearby as measured through Yelp. Applying our analysis pipeline to these null-treatments, we found that all of these effect estimates were close to zero. This demonstrated that our statistical analysis approach did not produce measurements that it was not supposed to measure; that is, discriminant validity. Estimates are based on matching experiments controlling for all but one treatment variable, across $N = 4911$ matched pairs of zip codes (Methods). Measure of centre reflects mean values; error bars correspond to 95% bootstrap confidence intervals (Methods). 14

List of Tables

1 Estimated precision of food entry classifiers. 6

2 Outcome measures calculated at the zip code level for the **9,822** zip codes in our study, spanning **1,164,926** participants. 8
3 Demographic statistics for our study compared with nationally representative survey data. (*) indicates statistics calculated at the zip code level. 9
4 Effect sizes of all top/bottom half matching experiments (Fig. 4).P values (one-sided, unadjusted) are computed through bootstrapping; precise p-values cannot be provided for any $p < 0.001$ due to the computational complexity involved in bootstrapping (N=1000) (Methods). 10
5 Effect sizes of all ethnicity-specific top/bottom half matching experiments (Fig. 5). P values (one-sided, unadjusted) are computed through bootstrapping; precise p-values cannot be provided for any $p < 0.001$ due to the computational complexity involved in bootstrapping (N=1000) (Methods). 11
6 Effect sizes of all top/bottom quartiles matching experiments (Fig. 2). P values (one-sided, unadjusted) are computed through bootstrapping; precise p-values cannot be provided for any $p < 0.001$ due to the computational complexity involved in bootstrapping (N=1000) (Methods). 12
7 Effect sizes of all null experiments to demonstrate discriminant validity. P values (one-sided, unadjusted) are computed through bootstrapping; precise p-values cannot be provided for any $p < 0.001$ due to the computational complexity involved in bootstrapping (N=1000) (Methods). 15
8 USA fast food restaurants table used to classify participant food entries as fast food[1]. A list of popular pizza chains from the USA was appended to the list[2]. . . . 16
9 USA soda list used to classify participant food entries as sugary sodas. The list was constructed using a list of America's best-selling brands of Soda[3], in addition to the generic terms such "Root Beer" and "Coca". 17

10 50 random food entries labeled positive by our fresh fruit or vegetable classifier as a fresh fruit or vegetable (* signifies a misclassification). The precision for our classifier is 92% based on annotations from a trained nutritionist, using the USDA MyPlate food groups to operationalize fruits and vegetables. The only divergence from USDA MyPlate is that we intentionally excluded fruit and vegetable juices, taking a conservative approach to estimating diet healthiness (See "Details on outcome measures" in Supplementary Methods). 18
11 50 random food entries labeled positive by our fast food classifier as a fast food (* signifies a misclassification). We defined fast food according to standard limited service-based definitions from prior work [4]. According to this definition, our classifier achieves a precision of 86%. We could have alternatively used a definition of fast food based on nutrient density, which is challenging because meal-level health is ill-defined (e.g., researchers disagree on whether a salad at McDonald's with heavy Caesar dressing should be considered as fast food [4]). Based on annotations from a trained nutritionist using this more conservative definition, the precision for our classifier would still be 80%. 19
12 50 random food entries labeled positive by our soda classifier as a soda (* signifies a misclassification). 20
13 Zipcode-level Pearson correlations (R) between our four outcome variables (Fresh F&V, Fast Food, Soda, % Affected by Overweight or Obesity) and gender/age. Note that all correlations are very small, indicating that gender and age do not explain much variance of zip code level food consumption and BMI status. Therefore, we do not include gender and age covariates in our matching-based statistical analysis. However, we confirmed that additionally controlling for these two factors led to highly similar results and findings (Pearson Correlation R=0.95). 21
14 Summary of High Income (MedianFamilyIncome > Median) matching experiment.[1] 22
15 Summary of High Grocery (grocery store access > Median) matching experiment . 23
16 Summary of High Educational Attainment (% College Degrees > Median) matching experiment 24

17 Summary of Low Fast Food (% Yelp Fast Food < Median) matching experiment . 25
18 Summary of High Income (MedianFamilyIncome > 75th Percentile) matching experiment . . . 26
19 Summary of High Grocery (grocery store access > 75th Percentile) matching experiment . . . 27
20 Summary of High Educational Attainment (% College Degrees > 75th Percentile) matching experiment. **Note:** Treatment samples unmatched due to 0.35 STD caliper used to ensure 0.25 SMD balancing constraint. . . . 28
21 Summary of Low Fast Food (% Yelp Fast Food < 25th Percentile) matching experiment. **Note:** Treatment samples unmatched due to 1.6 STD caliper used to ensure 0.25 SMD balancing constraint. . . . 29
22 Summary of Low Countertop Installation Services (% Yelp Countertop Installers < Median) matching experiment . . . 30
23 Summary of Low Electronics Stores (% Yelp Electronics Stores < Median) matching experiment . . . 31
24 Summary of Low Waterproofing Services (% Yelp Waterproofing Services < Median) matching experiment . . . 32
25 Summary of Black-majority Zip Code High Income (MedianFamilyIncome > Median) matching experiment . . . 33
26 Summary of Hispanic-majority Zip Code High Income (MedianFamilyIncome > Median) matching experiment . . . 34
27 Summary of white-majority Zip Code High Income (MedianFamilyIncome > Median) matching experiment . . . 35
28 Summary of Black-majority Zip Code High Grocery (grocery store access > Median) matching experiment . . . 36
29 Summary of Hispanic-majority Zip Code High Grocery (grocery store access > Median) matching experiment . . . 37
30 Summary of white-majority Zip Code High Grocery (grocery store access > Median) matching experiment . . . 38

31 Summary of Black-majority Zip Code High Educational Attainment (% College Degrees > Median) matching experiment 39
32 Summary of Hispanic-majority Zip Code High Educational Attainment (% College Degrees > Median) matching experiment 40
33 Summary of white-majority Zip Code High Educational Attainment (% College Degrees > Median) matching experiment. **Note:** Treatment samples unmatched due to 2.1 STD caliper used to ensure 0.25 SMD balancing constraint. 41
34 Summary of Black-majority Zip Code Low Fast Food (% Yelp Fast Food < Median) matching experiment 42
35 Summary of Hispanic-majority Zip Code Low Fast Food (% Yelp Fast Food < Median) matching experiment 43
36 Summary of white-majority Zip Code Low Fast Food (% Yelp Fast Food < Median) matching experiment 44

Supplementary Table 1: Estimated precision of food entry classifiers.

Classifier	Estimated precision	Raw data
Fresh Fruit & Vegetable	92%	Table 10
Fast Food	86%	Table 11
Soda	96%	Table 12

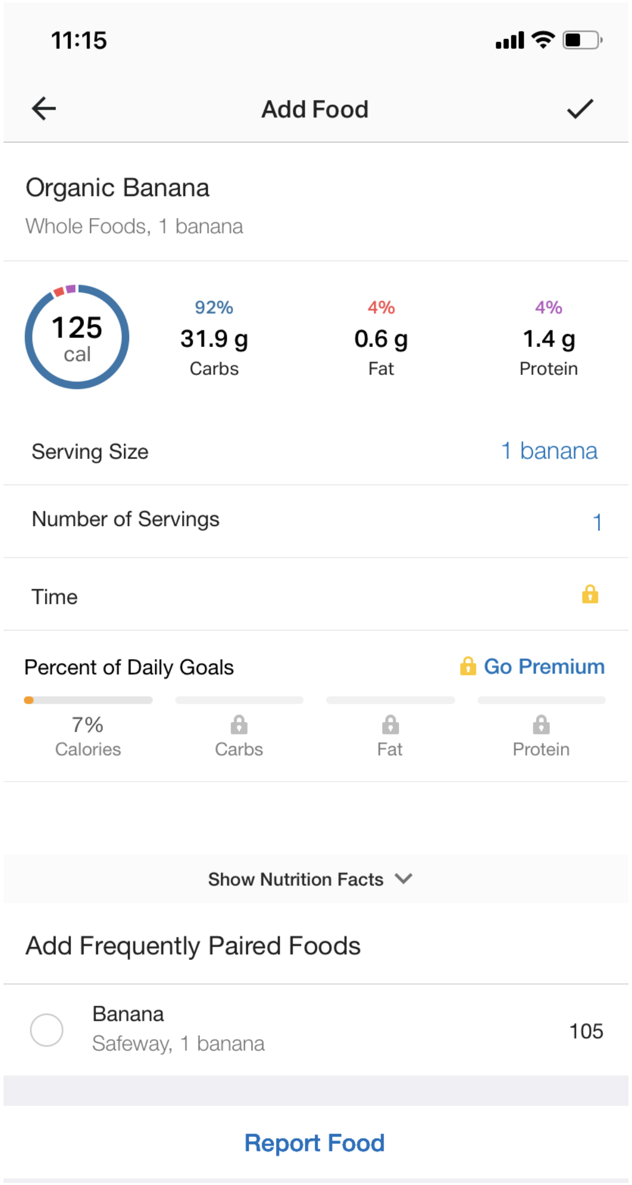

Supplementary Figure 1: **MyFitnessPal food logging app interface.** A screenshot of the MyFitnessPal app, showing the information collected for each food entry.

Supplementary Table 2: Outcome measures calculated at the zip code level for the **9,822** zip codes in our study, spanning **1,164,926** participants.

	# Participants	F&V Entries per Day	Soda Entries per Day	Fast Food Entries per Day	BMI	% Affected by Overweight or Obesity	% Affected by Obesity
mean	118.6	0.61	0.04	0.39	28.8	69	35
median	90	0.60	0.04	0.38	28.8	70	35
std	91.5	0.11	0.02	0.10	1.6	10	11
min	30	0.25	0.001	0.12	23.2	17	2
max	1262	1.29	0.17	0.91	36.9	100	80

Supplementary Table 3: Demographic statistics for our study compared with nationally representative survey data. (*) indicates statistics calculated at the zip code level.

Source	BMI	% Affected by Overweight or Obesity	% Affected by Obesity	Median Age	Gender	Median Family Income*	College*	Ethnicity*
Our Study	28.5	67.8%	32.8%	36	74% Female	$ 76,563	33.7%	68.3% white
Nat.l Avg.	29.4[5]	71.6%[6]	39.8%[6]	38.2[7]	50.5% Female[8]	$ 59,039[9]	33.4%[10]	61.3% white[11]

Supplementary Table 4: Effect sizes of all top/bottom half matching experiments (Fig. 4).P values (one-sided, unadjusted) are computed through bootstrapping; precise p-values cannot be provided for any $p < 0.001$ due to the computational complexity involved in bootstrapping (N=1000) (Methods).

Treatment	Outcome	% Difference	Ctrl. Mean	Trt. Mean	P (bootstrapping)
High Income	Fresh F&V Consumption	3.265	0.631	0.652	< 0.001
High Income	Fast Food Consumption	-6.772	0.367	0.342	< 0.001
High Income	Soda Consumption	-8.589	0.025	0.023	< 0.001
High Income	BMI	-0.335	28.077	27.983	< 0.001
High Income	% Affected by Overweight or Obesity	0.643	0.647	0.651	0.006
High Educational Attainment	Fresh F&V Consumption	9.180	0.602	0.657	< 0.001
High Educational Attainment	Fast Food Consumption	-8.457	0.371	0.340	< 0.001
High Educational Attainment	Soda Consumption	-13.834	0.026	0.022	< 0.001
High Educational Attainment	BMI	-5.053	29.266	27.787	< 0.001
High Educational Attainment	% Affected by Overweight or Obesity	-13.100	0.734	0.638	< 0.001
Low Fast Food	Fresh F&V Consumption	5.311	0.612	0.645	< 0.001
Low Fast Food	Fast Food Consumption	-6.176	0.370	0.347	< 0.001
Low Fast Food	Soda Consumption	-13.340	0.026	0.023	< 0.001
Low Fast Food	BMI	-0.335	28.467	28.371	< 0.001
Low Fast Food	% Affected by Overweight or Obesity	-1.474	0.679	0.669	< 0.001
High Grocery	Fresh F&V Consumption	3.437	0.606	0.627	< 0.001
High Grocery	Fast Food Consumption	-7.581	0.390	0.361	< 0.001
High Grocery	Soda Consumption	-6.363	0.027	0.025	< 0.001
High Grocery	BMI	-0.698	28.839	28.638	< 0.001
High Grocery	% Affected by Overweight or Obesity	-2.437	0.699	0.682	< 0.001

Supplementary Table 5: Effect sizes of all ethnicity-specific top/bottom half matching experiments (Fig. 5). P values (one-sided, unadjusted) are computed through bootstrapping; precise p-values cannot be provided for any $p < 0.001$ due to the computational complexity involved in bootstrapping (N=1000) (Methods).

Ethnicity	Treatment	Outcome	% Difference	Ctrl. Mean	Trt. Mean	P-value (bootstrapping)
Black	High Income	Fresh F&V Consumption	-6.497	0.607	0.567	0.004
Black	High Income	Fast Food Consumption	5.464	0.403	0.425	0.015
Black	High Income	Soda Consumption	14.153	0.025	0.029	0.061
Black	High Income	BMI	3.695	29.834	30.936	< 0.001
Black	High Income	% Affected by Overweight or Obesity	8.101	0.750	0.811	< 0.001
Black	High Educational Attainment	Fresh F&V Consumption	11.243	0.558	0.620	< 0.001
Black	High Educational Attainment	Fast Food Consumption	-7.613	0.424	0.391	0.002
Black	High Educational Attainment	Soda Consumption	-5.623	0.027	0.025	0.191
Black	High Educational Attainment	BMI	-5.512	31.362	29.634	< 0.001
Black	High Educational Attainment	% Affected by Overweight or Obesity	-11.481	0.829	0.734	< 0.001
Black	Low Fast Food	Fresh F&V Consumption	7.024	0.543	0.582	< 0.001
Black	Low Fast Food	Fast Food Consumption	-12.043	0.473	0.416	< 0.001
Black	Low Fast Food	Soda Consumption	-8.994	0.029	0.026	0.002
Black	Low Fast Food	BMI	0.506	30.776	30.931	0.153
Black	Low Fast Food	% Affected by Overweight or Obesity	3.062	0.775	0.799	0.001
Black	High Grocery	Fresh F&V Consumption	10.230	0.527	0.581	< 0.001
Black	High Grocery	Fast Food Consumption	-12.642	0.478	0.418	< 0.001
Black	High Grocery	Soda Consumption	-5.426	0.029	0.027	0.060
Black	High Grocery	BMI	-3.795	31.966	30.753	< 0.001
Black	High Grocery	% Affected by Overweight or Obesity	-8.960	0.861	0.783	< 0.001
Hispanic	High Educational Attainment	Fresh F&V Consumption	8.859	0.575	0.626	< 0.001
Hispanic	High Educational Attainment	Fast Food Consumption	-11.902	0.385	0.339	< 0.001
Hispanic	High Educational Attainment	Soda Consumption	-16.521	0.026	0.021	< 0.001
Hispanic	High Educational Attainment	BMI	-5.949	29.738	27.969	< 0.001
Hispanic	High Educational Attainment	% Affected by Overweight or Obesity	-13.697	0.758	0.654	< 0.001
Hispanic	High Income	Fresh F&V Consumption	5.706	0.556	0.588	0.012
Hispanic	High Income	Fast Food Consumption	-3.314	0.393	0.380	0.140
Hispanic	High Income	Soda Consumption	1.592	0.024	0.025	0.397
Hispanic	High Income	BMI	0.289	28.997	29.080	0.280
Hispanic	High Income	% Affected by Overweight or Obesity	-0.039	0.722	0.722	0.492
Hispanic	Low Fast Food	Fresh F&V Consumption	1.501	0.562	0.570	0.083
Hispanic	Low Fast Food	Fast Food Consumption	-5.920	0.423	0.398	< 0.001
Hispanic	Low Fast Food	Soda Consumption	-0.595	0.029	0.028	0.434
Hispanic	Low Fast Food	BMI	-0.172	29.750	29.699	0.291
Hispanic	Low Fast Food	% Affected by Overweight or Obesity	-1.800	0.763	0.750	0.004
Hispanic	High Grocery	Fresh F&V Consumption	7.351	0.525	0.563	< 0.001
Hispanic	High Grocery	Fast Food Consumption	-7.212	0.443	0.411	< 0.001
Hispanic	High Grocery	Soda Consumption	5.137	0.028	0.029	0.029
Hispanic	High Grocery	BMI	-1.518	30.358	29.898	< 0.001
Hispanic	High Grocery	% Affected by Overweight or Obesity	-3.492	0.791	0.763	< 0.001
white	High Income	Fresh F&V Consumption	2.182	0.643	0.657	< 0.001
white	High Income	Fast Food Consumption	-5.063	0.359	0.341	< 0.001
white	High Income	Soda Consumption	-8.827	0.025	0.023	< 0.001
white	High Income	BMI	0.486	27.759	27.894	< 0.001
white	High Income	% Affected by Overweight or Obesity	3.261	0.625	0.646	< 0.001
white	High Educational Attainment	Fresh F&V Consumption	9.690	0.600	0.658	< 0.001
white	High Educational Attainment	Fast Food Consumption	-5.878	0.363	0.342	< 0.001
white	High Educational Attainment	Soda Consumption	-7.712	0.025	0.023	< 0.001
white	High Educational Attainment	BMI	-4.100	28.955	27.768	< 0.001
white	High Educational Attainment	% Affected by Overweight or Obesity	-11.105	0.717	0.638	< 0.001
white	Low Fast Food	Fresh F&V Consumption	6.028	0.625	0.663	< 0.001
white	Low Fast Food	Fast Food Consumption	-6.611	0.359	0.335	< 0.001
white	Low Fast Food	Soda Consumption	-13.542	0.025	0.022	< 0.001
white	Low Fast Food	BMI	-0.644	28.147	27.966	< 0.001
white	Low Fast Food	% Affected by Overweight or Obesity	-2.373	0.662	0.647	< 0.001
white	High Grocery	Fresh F&V Consumption	1.655	0.634	0.644	< 0.001
white	High Grocery	Fast Food Consumption	-5.004	0.368	0.349	< 0.001
white	High Grocery	Soda Consumption	-3.536	0.026	0.025	0.001
white	High Grocery	BMI	-0.104	28.239	28.209	0.082
white	High Grocery	% Affected by Overweight or Obesity	-1.373	0.665	0.656	< 0.001

Supplementary Table 6: Effect sizes of all top/bottom quartiles matching experiments (Fig. 2). P values (one-sided, unadjusted) are computed through bootstrapping; precise p-values cannot be provided for any $p < 0.001$ due to the computational complexity involved in bootstrapping (N=1000) (Methods).

Treatment	Outcome	% Difference	Trt. Mean	Ctrl. Mean	P-value (bootstrapping.)
High Income	Fresh F&V Consumption	6.157	0.641	0.680	< 0.001
High Income	Fast Food Consumption	-8.229	0.342	0.314	< 0.001
High Income	Soda Consumption	-8.815	0.021	0.019	< 0.001
High Income	BMI	0.697	27.183	27.373	< 0.001
High Income	% Affected by Overweight or Obesity	2.398	0.600	0.614	< 0.001
High Educational Attainment	Fresh F&V Consumption	8.111	0.613	0.663	< 0.001
High Educational Attainment	Fast Food Consumption	-7.231	0.369	0.343	< 0.001
High Educational Attainment	Soda Consumption	-8.930	0.025	0.023	< 0.001
High Educational Attainment	BMI	-5.188	29.049	27.542	< 0.001
High Educational Attainment	% Affected by Overweight or Obesity	-10.823	0.697	0.622	< 0.001
High Grocery	Fresh F&V Consumption	6.462	0.609	0.648	< 0.001
High Grocery	Fast Food Consumption	-11.714	0.380	0.335	< 0.001
High Grocery	Soda Consumption	-11.087	0.026	0.023	< 0.001
High Grocery	BMI	-0.847	28.622	28.380	< 0.001
High Grocery	% Affected by Overweight or Obesity	-3.244	0.687	0.665	< 0.001
Low Fast Food	Fresh F&V Consumption	9.424	0.610	0.668	< 0.001
Low Fast Food	Fast Food Consumption	-12.669	0.376	0.329	< 0.001
Low Fast Food	Soda Consumption	-23.220	0.026	0.020	< 0.001
Low Fast Food	BMI	-0.598	28.269	28.100	< 0.001
Low Fast Food	% Affected by Overweight or Obesity	-0.559	0.656	0.653	0.036

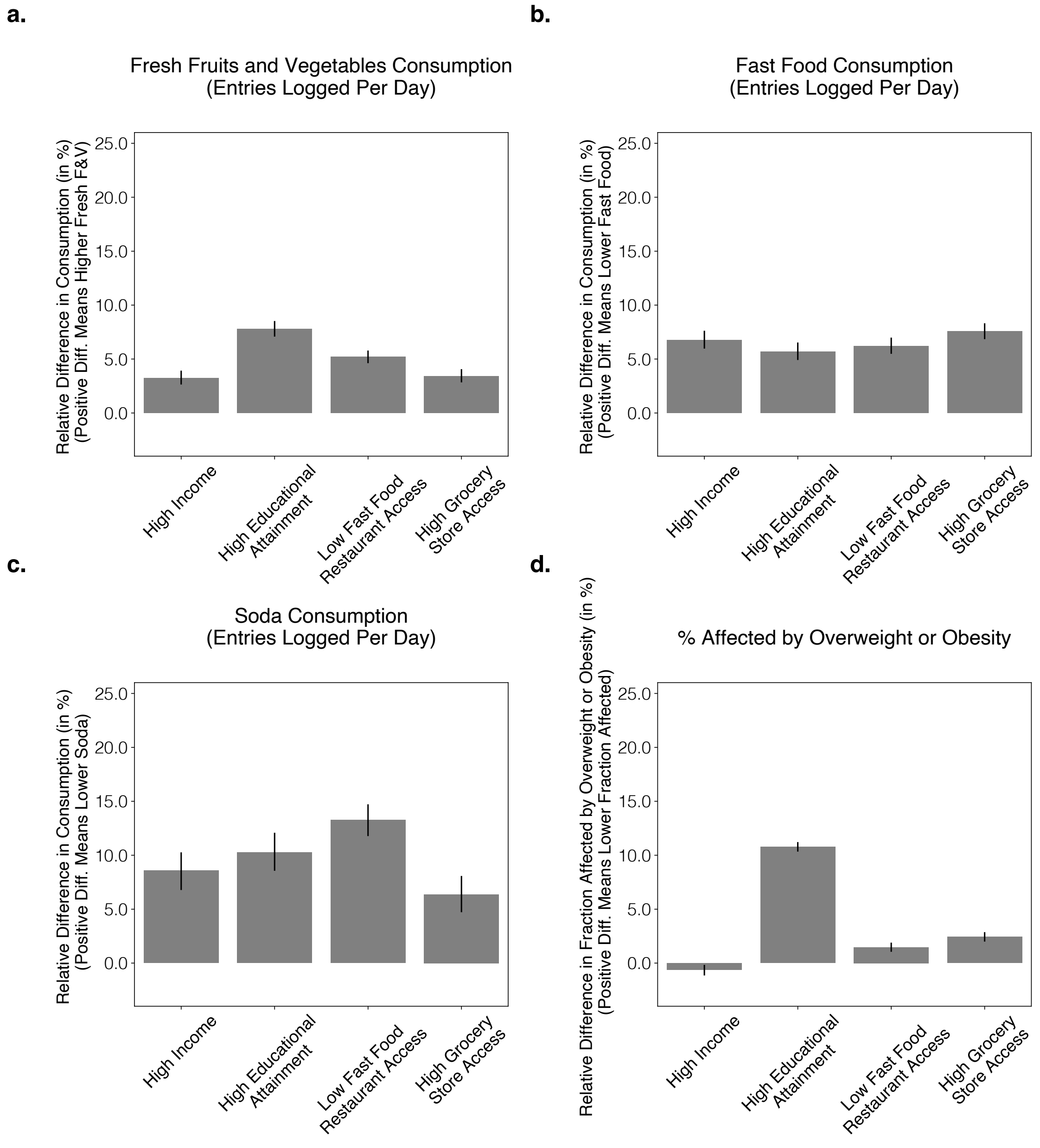

Supplementary Figure 2: Matching experiments using quartile instead of median split. Note the consistent and in many cases larger effect sizes compared to Figure 4. Estimates are based on matching experiments controlling for all but one treatment variable, across $N = 2456$ matched pairs of zip codes (Methods). Bar height corresponds to mean values; error bars correspond to 95% bootstrap confidence intervals (Methods).

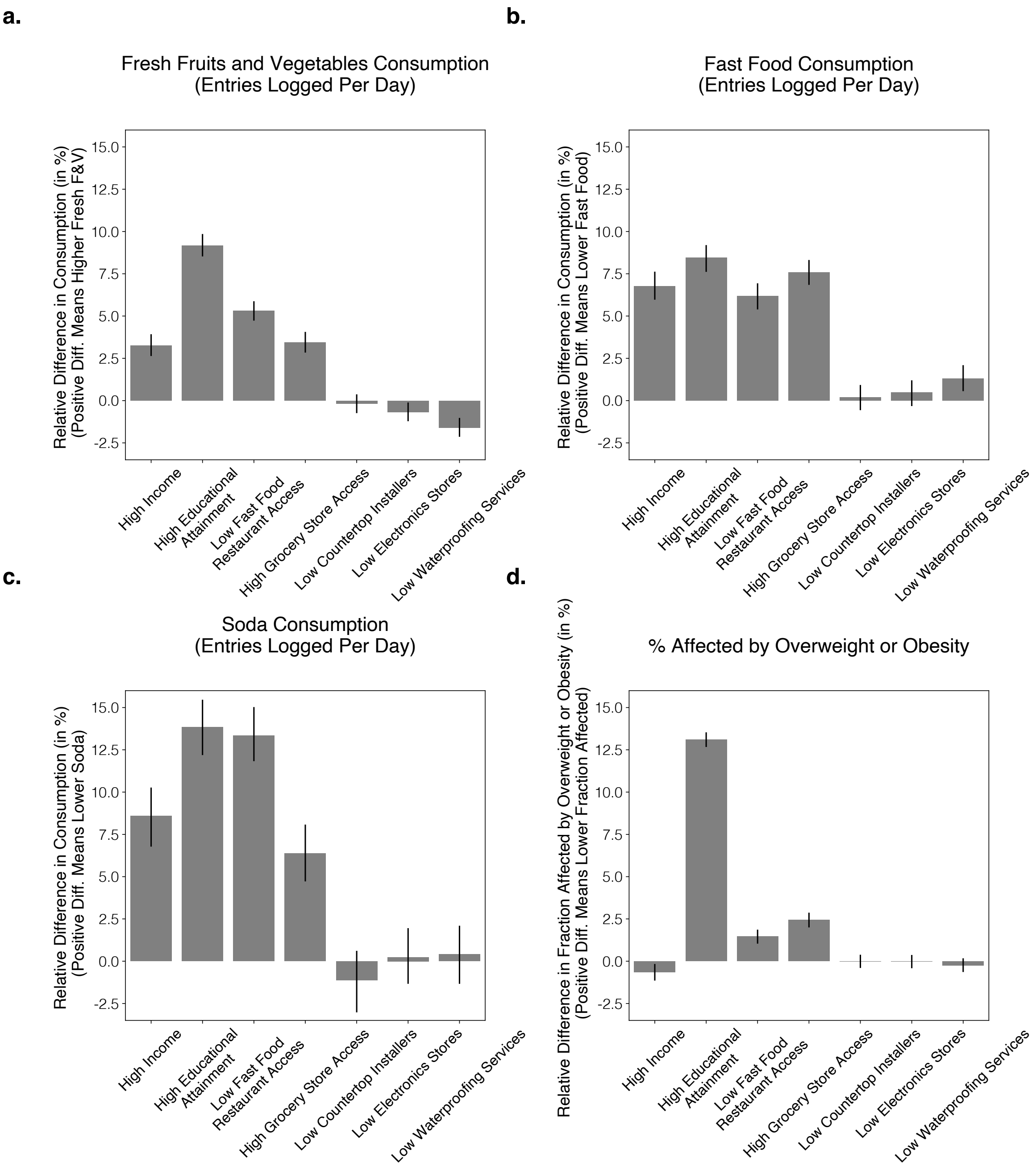

Supplementary Figure 3: **Demonstration of discriminant validity of statistical approach.** We measured the effect of null-treatments that should not have any impact on food consumption. We chose examples of null-treatments by selecting variables that had little correlation with study independent variables (income, educational attainment, grocery store access, fast food access) and were plausibly unrelated to food consumption. This selection process lead to use of the fraction of countertop installers, electronics stores, and waterproofing services nearby as measured through Yelp. Applying our analysis pipeline to these null-treatments, we found that all of these effect estimates were close to zero. This demonstrated that our statistical analysis approach did not produce measurements that it was not supposed to measure; that is, discriminant validity. Estimates are based on matching experiments controlling for all but one treatment variable, across $N = 4911$ matched pairs of zip codes (Methods). Measure of centre reflects mean values; error bars correspond to 95% bootstrap confidence intervals (Methods).

Supplementary Table 7: Effect sizes of all null experiments to demonstrate discriminant validity. P values (one-sided, unadjusted) are computed through bootstrapping; precise p-values cannot be provided for any $p < 0.001$ due to the computational complexity involved in bootstrapping (N=1000) (Methods).

Treatment	Outcome	% Difference	Ctrl. Mean	Trt. Mean	P (bootstrapping)
Low Countertop Svc.	Fresh F&V Consumption	-0.177	0.612	0.610	0.289
Low Countertop Svc.	Fast Food Consumption	-0.190	0.384	0.384	0.337
Low Countertop Svc.	Soda Consumption	1.131	0.027	0.028	0.101
Low Countertop Svc.	BMI	0.102	28.710	28.740	0.082
Low Countertop Svc.	% Affected by Overweight or Obesity	0.012	0.690	0.690	0.499
Low Electronics Stores	Fresh F&V Consumption	-0.681	0.609	0.605	0.011
Low Electronics Stores	Fast Food Consumption	-0.482	0.387	0.386	0.118
Low Electronics Stores	Soda Consumption	-0.241	0.028	0.028	0.359
Low Electronics Stores	BMI	0.006	28.906	28.908	0.467
Low Electronics Stores	% Affected by Overweight or Obesity	0.018	0.701	0.701	0.467
Low Waterproofing Svc.	Fresh F&V Consumption	-1.607	0.614	0.604	< 0.001
Low Waterproofing Svc.	Fast Food Consumption	-1.319	0.389	0.384	< 0.001
Low Waterproofing Svc.	Soda Consumption	-0.420	0.028	0.028	0.340
Low Waterproofing Svc.	BMI	0.046	28.786	28.799	0.274
Low Waterproofing Svc.	% Affected by Overweight or Obesity	0.239	0.694	0.695	0.133

Supplementary Table 8: USA fast food restaurants table used to classify participant food entries as fast food[1]. A list of popular pizza chains from the USA was appended to the list[2].

A&W Restaurants	Cinnabon	Red Burrito	Rogers Restaurants	Chuck E. Cheese's	Murphy's Pat's Pizza
Arby's	Claim Jumper	The Habit	Runza Saladworks	CiCi's Pizza Cottage	Patxi's Chicago Peter
Arctic Circle	Coco's	Halal Guys	Sbarro Schlotzsky's	Inn Dion's Discovery	Piper Pie Five
Arthurs	Cold Stone Creamery	Hardee's	Seattle's Best Shake	Zone Domino's	Pietro's Pizza Pizza
Atlanta Bread	Cookout	Huddle House	Shack Skyline Chili	Donatos	Corner Pizza Factory
Au Bon Pain	Copeland's	In-N-Out Burger	Sneaky Pete's Sonic	DoubleDave's East of	Pizza Fusion Pizza
Auntie Anne's	Old Country	Jack in the Box	Spangles Steak	Chicago Eatza	Hut Pizza King Pizza
Baja Fresh	Culver's	Jack's Family	Escape Steak 'n	Extreme Pizza	Inn Pizza My Heart
Bakers Square	Dairy Queen	Restaura	Shake Stir Crazy Sub	Fazoli's Fellini's	Pizza Patrón Pizza
Blimpies	el Tacos	ts Jersey Mike's Subs	Station II Subway	Fox's Frank Pepe	Ranch Pizza
Bojangles	DiBella's	Jimmy John's Jim's	Swensen's Swensons	Gatti's Gino's	Schmizza The Pizza
Boston Market	Dixie Chili and Deli	Restaurants Johnny	Taco Bell Taco	Giordano's	Studio Pizzeria Venti
Braum's	Don Pablo's	Rockets KFC	Bueno Taco Cabana	Godfather's	Regina Pizzeria
Burger Chef	Druther's	Kewpee Krispy	Taco John's Taco	Grimaldi's Grotto	Rocky Rococo
Burger King	Dunkin' Donuts	Kreme L&L	Mayo Taco Tico Taco	Pizza Happy Joe's	Rosati's Round Table
Burger Street	Eat'n Park	Hawaiian Barbecue	Time Twin Peaks	Happy's Hideaway	Pizza Russo's New
Burgerville	Eegee's	Lee Roy Selmon's	Umami Burger	Home Run Inn	York Pizze
Captain D's	El Chico	Lee's Famous Lion's	Wendy's Wetzel's	Hungry Howie's	ia Sal's Pizza
Carino's Italian Grill	El Pollo Loco	Choice Long John	Pretzels Whataburger	Hunt Brothers Imo's	Sammy's
Carl's Jr.	El Taco Tote	Silver's Luby's	white Castle	Pizza Jerry's Jet's	Sarpino's
Carrows	Elephant Bar	McDonald's Milo's	Wienerschnitzel	John's LaRosa's	Sbarro
Charley's Grilled	Elevation Burger	Moe's Mooyah Mr.	Wimpy Zaxby's	Ledo Little Caesars	Shakey's
Subs	Famous Dave's	Hero Mrs. Fields	Zero's Subs Zippy's	Lou Malnati's	Showbiz
Checkers	Farmer Boys	Mrs. Winner's	America's Incredible	Marco's Marion's	Sir Pizza
Cheeburger	Fatburger	Chicken	Arni's Aurelio's	Mark's Mazzio's	Snappy Tomato
Cheeburger	Firehouse Subs	Biscuits Naugles	Azzip Bearno's	Mellow Mushroom	Straw Hat
Chevys	Five Guys	Panera Bread Panda	Bertucci's Big	MOD Pizza	Toppers
Chicken Express	Freddy's	Express Penn Station	Mama's & Papa's	Monical's Mountain	Uncle Maddio's
Chick-fil-A	Freddies	Pita Pit Popeyes Port	Blackjack Blaze	Mike's Mr. Jim's	Unos
Chronic Tacos	Golden Chick	of Subs Potbell	Buddy's Bullwinkle's	Noble Roman's Old	Upper Crust Pizzeria
Chuck-A-Rama	Good Times	Quizno's Raising	California Pizza	Chicago Pacpizza	Valentino's
Church's	Great Steak	Cane's Rax Roast	Casey's General	Pagliacci Papa Gino's	Vocelli Pizza
Texas Chicken	Green Burrito	Beef Robeks Roy	Stores Cassano's	Papa John's Papa	Your Pie

Supplementary Table 9: USA soda list used to classify participant food entries as sugary sodas. The list was constructed using a list of America's best-selling brands of Soda[3], in addition to the generic terms such "Root Beer" and "Coca".

Pepsi	Dr Pepper	Root Beer
Coca Cola	Sprite	Coke
Mountain Dew	Fanta	Coca

Supplementary Table 10: 50 random food entries labeled positive by our fresh fruit or vegetable classifier as a fresh fruit or vegetable (* signifies a misclassification). The precision for our classifier is 92% based on annotations from a trained nutritionist, using the USDA MyPlate food groups to operationalize fruits and vegetables. The only divergence from USDA MyPlate is that we intentionally excluded fruit and vegetable juices, taking a conservative approach to estimating diet healthiness (See "Details on outcome measures" in Supplementary Methods).

Brand (empty if generic)	Description
Fresh	Pineapple Chunks
Apple	Apples
(none)	Squash - Zucchini, includes skin, cooked, boil...
Romaine Lettuce	3 Leaves
(none)	Lettuce - Green leaf, raw
Generic	Grapes - Red - Seedless
Generic	Medium Naval Orange
Generic (Fresh)	Broccali
(none)	Melons - Cantaloupe, raw
(none)	Apples - Raw, with skin
Baby	Tomato
Bagu Clementines	Clementines
(none)	Lettuce - Iceberg (includes crisphead types), raw
Ataulfo	Mango
Fruit	Fresh Purple Plum
Celery	Celery
Apples	Apples
(none)	Sweet potato - Raw, unprepared (Sweetpotato)
Honey*	Local Honey*
Asda	Seedless Green Grapes
Chiquita	Medium Banana
(none)	Spinach - Raw
Trader Joe's	Just Mango Slices, Dried Fruit
(none)	Broccoli - Frozen, chopped, unprepared
Chinese*	Sesame Chicken*
(none)	Celery - Raw
Strawberry	One
(none)	Grapes - Raw
(none)	Strawberries - Raw
(none)	Bananas - Raw
Fruit	Tangelo
Fresh Steamed	Zuchinni
(none)	Onion Slice
Cucumber	Cucumber
Fresh	Blueberries
Squash	Acorn
(none)	Broccoli - Cooked, boiled, drained, with salt
Artisan*	Granola*
Tomato	Tomato-Raw
(none)	Sweet potato - Cooked, baked in skin, without ...
Fresh Steamed	Carrots
(none)	Raspberries - Raw
Cuties	Mandrain Orange
(none) *	Nuts - Cashew nuts, raw*

Supplementary Table 11: 50 random food entries labeled positive by our fast food classifier as a fast food (* signifies a misclassification). We defined fast food according to standard limited service-based definitions from prior work [4]. According to this definition, our classifier achieves a precision of 86%. We could have alternatively used a definition of fast food based on nutrient density, which is challenging because meal-level health is ill-defined (e.g., researchers disagree on whether a salad at McDonald's with heavy Caesar dressing should be considered as fast food [4]). Based on annotations from a trained nutritionist using this more conservative definition, the precision for our classifier would still be 80%.

brand	description
braums*	mint chocolate chip*
marcos tonda*	chocolate*
marks	chick bacon swich
papa johns	sausage pizza thin crust
five guys	little cheeseburger
subway	6" turkey
subway	turkey breast, swiss, lettuce, onion, pickles,...
moes	Guacamole
subway	rotisserie chicken, lett, tom, bell pep, jalap...
subway	egg
mcdonalds	fruit and yogurt parfait without granola
potbelly	hot pepper s (from j ar)
dunkin donuts	dunkin donuts southwest gran breakfast burritto
mcdonalds	chicken nugg
johnsonville orginal breakfast link sausage*	sausage links*
subway	roadt beef
muscle blaze*	fat burner extreme*
moes	burrito5
mcdonalds	shamrock shake
subway	oven roasted chicken flatbread with american c...
kfc	original fillet burger
checkers	Fries
subway	oven roasted chick
papa johns	custom pizza
dominos	x large cheese pizza slice
zippys	Korean Fried Chicken
mcdonalds	fruit and maple oatmeal no cream
subway	6" on wheat , pepper jack cheese,lettuce,spina...
runza	Cheeseburger
marks and spencer*	super rice and quinoa*
taco bell	hot sauce
honest green tea mango wendys	tea Wendy's
shakeys	pork scratchings
subway	lite mayo
taco bell	taco sauce
jimmy johns s	unwhich double meat
chickfila house dressing	salad dressing13013
panera bread low fat chicken noodle soup	Chicken Soup
dunkin donuts	southwest steak buritto
krispy kreme	glazed choc glazed donut holes
ledo sladoled*	maximo šumsko voće*
marks and spencer*	biancoli spears*
churchs chicken	Biscuit
wendys	small unsweetened brewed iced tea

Supplementary Table 12: 50 random food entries labeled positive by our soda classifier as a soda (* signifies a misclassification).

brand	description
coca cola	coke mini 75oz
cocacola 24oz	regular cocacola
coca cola	sprite can
pepsi australia	pepsi cola 375ml can
aw root beer	10 calorie
coke	12 oz bottle
colamecos*	canneloni*
mountain dew kickstart drink	energy drink
pepsi max	20 oz bottle
12oz can of coke	coke
cocacola classic	coke 12 oz can
coca cola	can
cocacola company	full throttle energy drink citrus flavor
dr pepper	12oz can
coke	coke can 375 ml
dr pepper	regular soda
pepsi	regular
pepsi	regular soda 12oz can
mountain dew	mountian dew
dr pepper	bottle
svedka colada*	vodka*
pepsi	next
fanta	fanta orange 330ml can
pepsi	pepsi
coke	coke
dr pepper	12 fl oz 355 ml can
coca cola	169 fl oz 106pt 500 ml
pepsi	drink
pepsi	20 oz fountain soda
mountain dew	20oz bottle
coke	12oz can
cocacola company	coke 375ml can
sprite	can
pepsi	pepsi
dr pepper	20 oz bottle
dr pepper	soda 10 calories
pepsi	8oz
mountain dew	kickstart energizing orange citrus
dr pepper	small can
sprite	soda
coke	classic can
cocacola	12oz regular can coke
pepsi	75 oz can
coca cola	coke 20 oz bottle
coca cola	coke 20oz bottle
mountain dew	20 oz bottle
mountain dew	kickstart orange
pepsi max	12 fl oz can
cocacola	coke
coca cola	can of coke regular

Supplementary Table 13: Zipcode-level Pearson correlations (R) between our four outcome variables (Fresh F&V, Fast Food, Soda, % Affected by Overweight or Obesity) and gender/age. Note that all correlations are very small, indicating that gender and age do not explain much variance of zip code level food consumption and BMI status. Therefore, we do not include gender and age covariates in our matching-based statistical analysis. However, we confirmed that additionally controlling for these two factors led to highly similar results and findings (Pearson Correlation R=0.95).

	Gender	Age
Fresh F&V Entries/Day	-0.04	-0.02
Fast Food Entries/Day	-0.04	0.00
Soda Entries/Day	-0.02	0.02
% Affected by Overweight or Obesity	-0.06	0.12

Supplementary Table 14: Summary of High Income (MedianFamilyIncome > Median) matching experiment.[1]

(a) Sample sizes

	Control	Treated [2]
All	4911	4911 [3]
Matched	1358	4911 [4]
Unmatched	3553	0 [5]

(b) Summary of balance for matched data

	Means Treated	Means Control	SD Control	Mean Diff	Std. Mean Difference
distance[6]	0.75	0.75	0.26	0.00	0.01
Educational Attainment (% Without College Degree)	0.56	0.57	0.15	-0.01	-0.06
Grocery Distance (USDA lapophalfshare)	0.74	0.74	0.21	0.00	0.00
Yelp Fast Food %	0.06	0.06	0.06	0.00	-0.01
Median Family Income	97244.90	58991.91	8680.39	38253.00	4.41

[1] All matching summary tables were generated using the open source Genetic Matching library GenMatch in the R progamming language[12].

[2] Treated indicates the condition in the figure caption above is true (i.e., MedianFamilyIncome > Median) at the zip code level.

[3] This row indicates the total count of zip codes above and below the median before Genetic matching was applied.

[4] This row indicates the total count of zip codes above and below the median after Genetic matching was applied. The decrease in rows is because it is possible for one zip code in the Treated group to be matched to multiple zip codes in the Control group which are closest to it.

[5] Unmatched zipcodes are zip codes in the control group (below median) for which no match was found. In general there will be zero unmatched Treated samples, except when a caliper was applied in order to enforce the 0.25 Standardized Mean Difference constraint (e.g., Table e19).

[6] The average Mahalanobis distance for each sample in the treatment/control.

Supplementary Table 15: Summary of High Grocery (grocery store access > Median) matching experiment

(a) Sample sizes

	Control	Treated
All	4911	4911
Matched	2219	4911
Unmatched	2692	0

(b) Summary of balance for matched data

	Means Treated	Means Control	SD Control	Mean Diff	Std. Mean Difference
distance	0.59	0.58	0.19	0.00	0.01
Median Family Income	76817.91	77021.36	30133.94	-203.45	-0.01
Educational Attainment (% Without College Degree)	0.64	0.64	0.18	0.00	-0.01
Yelp Fast Food %	0.06	0.06	0.06	0.00	0.00
Grocery Distance (USDA lapophalfshare)	0.57	0.88	0.06	-0.31	-5.62

Supplementary Table 16: Summary of High Educational Attainment (% College Degrees > Median) matching experiment

(a) Sample sizes

	Control	Treated
All	4911	4911
Matched	1481	4911
Unmatched	3430	0

(b) Summary of balance for matched data

	Means Treated	Means Control	SD Control	Mean Diff	Std. Mean Difference
distance	0.73	0.73	0.27	0.00	0.01
Median Family Income	93203.36	88493.86	20530.36	4709.51	0.23
Grocery Distance (USDA lapophalfshare)	0.71	0.72	0.22	-0.01	-0.05
Yelp Fast Food %	0.06	0.06	0.06	0.00	0.02
Educational Attainment (% Without College Degree)	0.53	0.75	0.05	-0.22	-4.79

Supplementary Table 17: Summary of Low Fast Food (% Yelp Fast Food < Median) matching experiment

(a) Sample sizes

	Control	Treated
All	4911	4911
Matched	1910	4911
Unmatched	3001	0

(b) Summary of balance for matched data

	Means Treated	Means Control	SD Control	Mean Diff	Std. Mean Difference
distance	0.65	0.65	0.24	0.00	0.01
Median Family Income	86942.10	86597.30	31088.00	344.80	0.01
Educational Attainment (% Without College Degree)	0.60	0.61	0.18	0.00	-0.02
Grocery Distance (USDA lapophalfshare)	0.66	0.67	0.23	-0.01	-0.05
Yelp Fast Food %	0.03	0.11	0.06	-0.08	-1.35

Supplementary Table 18: Summary of High Income (MedianFamilyIncome > 75th Percentile) matching experiment

(a) Sample sizes

	Control	Treated
All	2456	2456
Matched	235	2456
Unmatched	2221	0

(b) Summary of balance for matched data

	Means Treated	Means Control	SD Control	Mean Diff	Std. Mean Difference
distance	0.91	0.91	0.19	0.00	0.01
Educational Attainment (% Without College Degree)	0.47	0.48	0.13	-0.02	-0.12
Grocery Distance (USDA lapophalfshare)	0.72	0.71	0.22	0.01	0.02
Yelp Fast Food (USDA lapophalfshare)	0.04	0.04	0.04	0.00	-0.06
Median Family Income	115169.38	47856.20	7989.15	67313.18	8.43

Supplementary Table 19: Summary of High Grocery (grocery store access > 75th Percentile) matching experiment

(a) Sample sizes

	Control	Treated
All	2456	2456
Matched	788	2456
Unmatched	1668	0

(b) Summary of balance for matched data

	Means Treated	Means Control	SD Control	Mean Diff	Std. Mean Difference
distance	0.69	0.67	0.22	0.01	0.05
Median Family Income	77550.55	77854.97	32233.91	-304.42	-0.01
Educational Attainment (% Without College Degree)	0.62	0.62	0.19	-0.01	-0.04
Yelp Fast Food (USDA lapophalfshare)	0.04	0.04	0.04	0.00	-0.01
Grocery Distance (USDA lapophalfshare)	0.42	0.94	0.03	-0.52	-15.48

Supplementary Table 20: Summary of High Educational Attainment (% College Degrees > 75th Percentile) matching experiment. **Note:** Treatment samples unmatched due to 0.35 STD caliper used to ensure 0.25 SMD balancing constraint.

(a) Sample sizes

	Control	Treated
All	2456	2456
Matched	262	1084
Unmatched	2194	1372

(b) Summary of balance for matched data

	Means Treated	Means Control	SD Control	Mean Diff	Std. Mean Difference
distance	0.77	0.76	0.29	0.01	0.04
Median Family Income	84490.50	82315.35	13441.21	2175.15	0.16
Grocery Distance (USDA lapophalfshare)	0.71	0.71	0.22	0.00	-0.01
Yelp Fast Food (USDA lapophalfshare)	0.06	0.06	0.06	0.00	0.03
Educational Attainment (% Without College Degree)	0.48	0.84	0.04	-0.36	-8.43

Supplementary Table 21: Summary of Low Fast Food (% Yelp Fast Food < 25th Percentile) matching experiment. **Note:** Treatment samples unmatched due to 1.6 STD caliper used to ensure 0.25 SMD balancing constraint.

(a) Sample sizes

	Control	Treated
All	2458	2455
Matched	543	2336
Unmatched	1915	119

(b) Summary of balance for matched data

	Means Treated	Means Control	SD Control	Mean Diff	Std. Mean Difference
distance	0.78	0.77	0.27	0.01	0.05
Median Family Income	90209.17	87850.88	25773.79	2358.29	0.09
Educational Attainment (% Without College Degree)	0.58	0.57	0.17	0.01	0.03
Grocery Distance (USDA lapophalfshare)	0.63	0.68	0.19	-0.04	-0.23
Yelp Fast Food (USDA lapophalfshare)	0.02	0.17	0.06	-0.15	-2.49

Supplementary Table 22: Summary of Low Countertop Installation Services (% Yelp Countertop Installers $<$ Median) matching experiment

(a) Sample sizes

	Control	Treated
All	4913	4909
Matched	2829	4909
Unmatched	2084	0

(b) Summary of balance for matched data

	Means Treated	Means Control	SD Control	Mean Diff	Std. Mean Difference
distance	0.50	0.50	0.05	0.00	0.01
Median Family Income	77255.32	77443.29	28361.82	-187.96	-0.01
Educational Attainment (% Without College Degree)	0.65	0.66	0.18	0.00	0.00
Grocery Distance (USDA lapophalfshare)	0.72	0.72	0.24	0.00	0.00
Yelp Fast Food %	0.09	0.09	0.08	0.00	0.01
Yelp Countertop Installers %	0.00	0.00	0.00	0.00	-1.61

Supplementary Table 23: Summary of Low Electronics Stores (% Yelp Electronics Stores < Median) matching experiment

(a) Sample sizes

	Control	Treated
All	4912	4910
Matched	2741	4910
Unmatched	2171	0

(b) Summary of balance for matched data

	Means Treated	Means Control	SD Control	Mean Diff	Std. Mean Difference
distance	0.52	0.52	0.09	0.00	0.00
Median Family Income	74567.18	74306.84	25820.76	260.34	0.01
Educational Attainment (% Without College Degree)	0.68	0.68	0.16	0.00	0.00
Grocery Distance (USDA lapophalfshare)	0.71	0.71	0.22	0.00	-0.01
Yelp Fast Food %	0.08	0.08	0.07	0.00	0.00
Yelp Electronics Stores %	0.00	0.00	0.00	0.00	-1.59

Supplementary Table 24: Summary of Low Waterproofing Services (% Yelp Waterproofing Services < Median) matching experiment

(a) Sample sizes

	Control	Treated
All	4913	4909
Matched	2876	4909
Unmatched	2037	0

(b) Summary of balance for matched data

	Means Treated	Means Control	SD Control	Mean Diff	Std. Mean Difference
distance	0.50	0.50	0.02	0.00	0.00
Median Family Income	76371.54	76438.89	28071.29	-67.35	0.00
Educational Attainment (% Without College Degree)	0.66	0.66	0.17	0.00	0.00
Grocery Distance (USDA lapophalfshare)	0.74	0.74	0.21	0.00	-0.01
Yelp Fast Food %	0.09	0.09	0.08	0.00	0.01
Yelp Waterproofing Services %	0.00	0.00	0.00	0.00	-1.48

Supplementary Table 25: Summary of Black-majority Zip Code High Income (MedianFamilyIncome > Median) matching experiment

(a) Sample sizes

	Control	Treated
All	317	42
Matched	30	42
Unmatched	287	0

(b) Summary of balance for matched data

	Means Treated	Means Control	SD Control	Mean Diff	Std. Mean Difference
distance	0.36	0.35	0.25	0.02	0.07
Educational Attainment (% Without College Degree)	0.65	0.65	0.09	-0.01	-0.08
Grocery Distance (USDA lapophalfshare)	0.68	0.67	0.22	0.01	0.02
Yelp Fast Food %	0.04	0.03	0.03	0.00	0.09
Median Family Income	88944.14	59779.41	8039.94	29164.73	3.63

Supplementary Table 26: Summary of Hispanic-majority Zip Code High Income (MedianFamily-Income > Median) matching experiment

(a) Sample sizes

	Control	Treated
All	482	67
Matched	51	67
Unmatched	431	0

(b) Summary of balance for matched data

	Means Treated	Means Control	SD Control	Mean Diff	Std. Mean Difference
distance	0.35	0.31	0.25	0.04	0.16
Educational Attainment (% Without College Degree)	0.70	0.72	0.12	-0.02	-0.16
Grocery Distance (USDA lapophalfshare)	0.62	0.63	0.20	-0.01	-0.05
Yelp Fast Food %	0.07	0.07	0.05	0.00	0.00
Median Family Income	82812.73	56050.33	8534.43	26762.40	3.14

Supplementary Table 27: Summary of white-majority Zip Code High Income (MedianFamilyIncome > Median) matching experiment

(a) Sample sizes

	Control	Treated
All	3421	4277
Matched	1023	4277
Unmatched	2398	0

(b) Summary of balance for matched data

	Means Treated	Means Control	SD Control	Mean Diff	Std. Mean Difference
distance	0.78	0.78	0.25	0.00	0.01
Educational Attainment (% Without College Degree)	0.55	0.56	0.15	-0.01	-0.07
Grocery Distance (USDA lapophalfshare)	0.76	0.76	0.19	0.00	-0.01
Yelp Fast Food %	0.06	0.06	0.06	0.00	-0.01
Median Family Income	98014.79	59878.82	8719.97	38135.97	4.37

Supplementary Table 28: Summary of Black-majority Zip Code High Grocery (grocery store access > Median) matching experiment

(a) Sample sizes

	Control	Treated
All	100	259
Matched	65	259
Unmatched	35	0

(b) Summary of balance for matched data

	Means Treated	Means Control	SD Control	Mean Diff	Std. Mean Difference
distance	0.78	0.77	0.15	0.01	0.08
Median Family Income	51410.82	51925.49	14357.87	-514.66	-0.04
Educational Attainment (% Without College Degree)	0.76	0.77	0.08	0.00	-0.03
Yelp Fast Food %	0.05	0.05	0.05	0.00	-0.08
Grocery Distance (USDA lapophalfshare)	0.53	0.86	0.04	-0.33	-9.43

Supplementary Table 29: Summary of Hispanic-majority Zip Code High Grocery (grocery store access > Median) matching experiment

(a) Sample sizes

	Control	Treated
All	78	471
Matched	66	471
Unmatched	12	0

(b) Summary of balance for matched data

	Means Treated	Means Control	SD Control	Mean Diff	Std. Mean Difference
distance	0.87	0.87	0.09	0.00	0.01
Median Family Income	52218.86	53068.49	13918.72	-849.63	-0.06
Educational Attainment (% Without College Degree)	0.82	0.83	0.08	-0.01	-0.12
Yelp Fast Food %	0.06	0.06	0.05	0.00	0.01
Grocery Distance (USDA lapophalfshare)	0.47	0.88	0.05	-0.41	-7.86

Supplementary Table 30: Summary of white-majority Zip Code High Grocery (grocery store access > Median) matching experiment

(a) Sample sizes

	Control	Treated
All	4494	3204
Matched	1741	3204
Unmatched	2753	0

(b) Summary of balance for matched data

	Means Treated	Means Control	SD Control	Mean Diff	Std. Mean Difference
distance	0.51	0.50	0.19	0.00	0.02
Median Family Income	84030.48	84275.98	31002.90	-245.50	-0.01
Educational Attainment (% Without College Degree)	0.59	0.59	0.18	0.00	-0.01
Yelp Fast Food %	0.07	0.07	0.07	0.00	0.00
Grocery Distance (USDA lapophalfshare)	0.62	0.89	0.05	-0.27	-4.92

Supplementary Table 31: Summary of Black-majority Zip Code High Educational Attainment (% College Degrees > Median) matching experiment

(a) Sample sizes

	Control	Treated
All	285	74
Matched	48	74
Unmatched	237	0

(b) Summary of balance for matched data

	Means Treated	Means Control	SD Control	Mean Diff	Std. Mean Difference
distance	0.46	0.45	0.29	0.01	0.02
Median Family Income	70932.69	71048.45	22123.91	-115.77	-0.01
Grocery Distance (USDA lapophalfshare)	0.59	0.60	0.28	-0.01	-0.04
Yelp Fast Food %	0.04	0.04	0.04	0.00	0.00
Educational Attainment (% Without College Degree)	0.62	0.77	0.04	-0.15	-3.39

Supplementary Table 32: Summary of Hispanic-majority Zip Code High Educational Attainment (% College Degrees > Median) matching experiment

(a) Sample sizes

	Control	Treated
All	488	61
Matched	43	61
Unmatched	445	0

(b) Summary of balance for matched data

	Means Treated	Means Control	SD Control	Mean Diff	Std. Mean Difference
distance	0.33	0.28	0.22	0.05	0.21
Median Family Income	71569.13	67609.51	16348.80	3959.62	0.24
Grocery Distance (USDA lapophalfshare)	0.51	0.52	0.27	-0.01	-0.04
Yelp Fast Food %	0.05	0.05	0.04	0.00	-0.06
Educational Attainment (% Without College Degree)	0.61	0.80	0.05	-0.19	-3.79

Supplementary Table 33: Summary of white-majority Zip Code High Educational Attainment (% College Degrees > Median) matching experiment. **Note:** Treatment samples unmatched due to 2.1 STD caliper used to ensure 0.25 SMD balancing constraint.

(a) Sample sizes

	Control	Treated
All	3491	4207
Matched	1114	4102
Unmatched	2377	105

(b) Summary of balance for matched data

	Means Treated	Means Control	SD Control	Mean Diff	Std. Mean Difference
distance	0.75	0.75	0.26	0.00	0.01
Median Family Income	92654.95	88357.13	17926.24	4297.82	0.24
Grocery Distance (USDA lapophalfshare)	0.74	0.76	0.17	-0.01	-0.07
Yelp Fast Food %	0.07	0.07	0.06	0.00	0.00
Educational Attainment (% Without College Degree)	0.53	0.75	0.05	-0.22	-4.59

Supplementary Table 34: Summary of Black-majority Zip Code Low Fast Food (% Yelp Fast Food $<$ Median) matching experiment

(a) Sample sizes

	Control	Treated
All	100	259
Matched	70	259
Unmatched	30	0

(b) Summary of balance for matched data

	Means Treated	Means Control	SD Control	Mean Diff	Std. Mean Difference
distance	0.77	0.76	0.16	0.00	0.03
Median Family Income	54812.73	54366.39	18020.16	446.34	0.02
Educational Attainment (% Without College Degree)	0.75	0.74	0.10	0.01	0.09
Grocery Distance (USDA lapophalfshare)	0.57	0.60	0.22	-0.02	-0.11
Yelp Fast Food %	0.03	0.11	0.07	-0.08	-1.21

Supplementary Table 35: Summary of Hispanic-majority Zip Code Low Fast Food (% Yelp Fast Food < Median) matching experiment

(a) Sample sizes

	Control	Treated
All	252	297
Matched	135	297
Unmatched	117	0

(b) Summary of balance for matched data

	Means Treated	Means Control	SD Control	Mean Diff	Std. Mean Difference
distance	0.61	0.61	0.19	0.00	0.02
Median Family Income	52130.57	51853.43	13411.40	277.14	0.02
Educational Attainment (% Without College Degree)	0.81	0.82	0.09	-0.01	-0.11
Grocery Distance (USDA lapophalfshare)	0.45	0.45	0.26	0.00	-0.01
Yelp Fast Food %	0.03	0.10	0.06	-0.07	-1.20

Supplementary Table 36: Summary of white-majority Zip Code Low Fast Food (% Yelp Fast Food $<$ Median) matching experiment

(a) Sample sizes

	Control	Treated
All	4188	3510
Matched	1362	3510
Unmatched	2826	0

(b) Summary of balance for matched data

	Means Treated	Means Control	SD Control	Mean Diff	Std. Mean Difference
distance	0.65	0.65	0.26	0.00	0.01
Median Family Income	95359.35	94854.08	29365.91	505.27	0.02
Educational Attainment (% Without College Degree)	0.56	0.57	0.17	0.00	-0.01
Grocery Distance (USDA lapophalfshare)	0.71	0.72	0.20	-0.01	-0.05
Yelp Fast Food %	0.03	0.11	0.06	-0.08	-1.37

Bibliography

1. Wikipedia. List of fast food restaurant chains — Wikipedia, the free encyclopedia. http://en.wikipedia.org/w/index.php?title=List%20of%20fast%20food%20restaurant%20chains&oldid=933158460 (2017). [Online; accessed 01-September-2017].

2. Wikipedia. List of restaurant chains in the United States — Wikipedia, the free encyclopedia. http://en.wikipedia.org/w/index.php?title=List%20of%20restaurant%20chains%20in%20the%20United%20States&oldid=930612669 (2017). [Online; accessed 01-September-2017].

3. Hartlaub, P. Sweet! america's top 10 brands of soda (2017).

4. Fleischhacker, S. E., Evenson, K. R., Rodriguez, D. A. & Ammerman, A. S. A systematic review of fast food access studies. *Obesity Reviews* **12**, e460–e471 (2011).

5. CDC. Healthy weight, overweight, and obesity among u.s. adults (2018). URL https://www.cdc.gov/nchs/data/nhsr/nhsr122-508.pdf.

6. CDC. Obesity and overweight (2016). URL https://www.cdc.gov/nchs/fastats/obesity-overweight.htm.

7. Wilson, J. Americans getting older, new census figures show (2019). URL https://thehill.com/homenews/state-watch/449505-americans-getting-older-new-census-figures-show.

8. Worldbank. Population, female (% of total population) (2019). URL https://data.worldbank.org/indicator/SP.POP.TOTL.FE.ZS.

9. USCB. Income, poverty and health insurance coverage in the united states: 2016 (2017). URL https://www.census.gov/newsroom/press-releases/2017/income-povery.html.

10. Wilson, J. Census: More americans have college degrees than ever before (2017). URL https://thehill.com/homenews/state-watch/326995-census-more-americans-have-college-degrees-than-ever-before.

11. USCB. Quickfacts (2017). URL https://www.census.gov/quickfacts/fact/table/US/PST045219.

12. Sekhon, J. S. Multivariate and propensity score matching software with automated balance optimization: the matching package for r. *Journal of Statistical Software, Forthcoming* (2008).
