# Supplementary material for: Large-scale diet tracking data reveal disparate associations between food environment and diet
Source: Nat Commun. 2022 Jan 18;13:267. doi: 10.1038/s41467-021-27522-y (PMC8766578; doi:10.1038/s41467-021-27522-y)
Supplement: Supplementary file 2 — Reporting Summary [file 41467_2021_27522_MOESM2_ESM.pdf]

nature research

Corresponding author(s): Tim Althoff

Last updated by author(s): Oct 25, 2021

Reporting Summary

Nature Research wishes to improve the reproducibility of the work that we publish. This form provides structure for consistency and transparency in reporting. For further information on Nature Research policies, see our Editorial Policies and the Editorial Policy Checklist.

Statistics

For all statistical analyses, confirm that the following items are present in the figure legend, table legend, main text, or Methods section.

n/a	Confirmed	
☐	☒	The exact sample size (n) for each experimental group/condition, given as a discrete number and unit of measurement
☐	☒	A statement on whether measurements were taken from distinct samples or whether the same sample was measured repeatedly
☐	☒	The statistical test(s) used AND whether they are one- or two-sided<br>*Only common tests should be described solely by name; describe more complex techniques in the Methods section.*
☐	☒	A description of all covariates tested
☐	☒	A description of any assumptions or corrections, such as tests of normality and adjustment for multiple comparisons
☐	☒	A full description of the statistical parameters including central tendency (e.g. means) or other basic estimates (e.g. regression coefficient) AND variation (e.g. standard deviation) or associated estimates of uncertainty (e.g. confidence intervals)
☐	☒	For null hypothesis testing, the test statistic (e.g. F, t, r) with confidence intervals, effect sizes, degrees of freedom and P value noted<br>*Give P values as exact values whenever suitable.*
☒	☐	For Bayesian analysis, information on the choice of priors and Markov chain Monte Carlo settings
☒	☐	For hierarchical and complex designs, identification of the appropriate level for tests and full reporting of outcomes
☐	☒	Estimates of effect sizes (e.g. Cohen's d, Pearson's r), indicating how they were calculated

Our web collection on statistics for biologists contains articles on many of the points above.

Software and code

Policy information about availability of computer code

Data collection

Provide a description of all commercial, open source and custom code used to collect the data in this study, specifying the version used OR state that no software was used.

Data analysis

We use Python 2.7.5 and R 3.5.0 programming languages and standard software packages including:

Python:
matplotlib 2.2.2
pandas 0.23.0
statsmodels 0.9.0
seaborn 0.9.0
scipy 1.2.0
requests 2.24.0
urllib3 1.23

R:
MatchIt MatchIt 3.0.2
rgenoud rgenoud 5.8-2.0
dplyr.1 dplyr 0.7.6
knitr.1 knitr 1.15.1

For manuscripts utilizing custom algorithms or software that are central to the research but not yet described in published literature, software must be made available to editors and reviewers. We strongly encourage code deposition in a community repository (e.g. GitHub). See the Nature Research guidelines for submitting code & software for further information.

Data

Policy information about availability of data

All manuscripts must include a data availability statement. This statement should provide the following information, where applicable:

- Accession codes, unique identifiers, or web links for publicly available datasets
- A list of figures that have associated raw data
- A description of any restrictions on data availability

Data was collected from MyFitnessPal smartphone app (food logging), from Yelp (local businesses), and from US Census data (demographics). All data at zipcode level will be released publicly with publication. See the paper for access information.

Field-specific reporting

Please select the one below that is the best fit for your research. If you are not sure, read the appropriate sections before making your selection.

☐ Life sciences ☒ Behavioural & social sciences ☐ Ecological, evolutionary & environmental sciences

For a reference copy of the document with all sections, see nature.com/documents/nr-reporting-summary-flat.pdf

Behavioural & social sciences study design

All studies must disclose on these points even when the disclosure is negative.

Study description	Quantitative, retrospective observational study of a sample of 1,164,926 participants using the MyFitnessPal app across 9,822 U.S. zip codes logging 2.3 billion consumed foods. This data was joined with Census information (demographics) and Yelp data on local businesses. A matching-based study was conducted to study the independent contributions of fast food and grocery access, income and education to diet health outcomes.
Research sample	Participants were users of the My-FitnessPal smartphone application. All data was anonymized and aggregated at the zip code level. Sample was a non-representative sample; for comparison with U.S. population see paper.
Sampling strategy	Retrospective observational study, where sample size was determined by the size of the shared sample from MyFitnessPal.
Data collection	Data was collected from MyFitnessPal smartphone app (food logging), from Yelp (local businesses), and from US Census data (demographics). All data was anonymized and aggregated at the zip code level.
Timing	7-year observation period between 2010 and 2016
Data exclusions	All participants in our sample used the app for at least 10 days.
Non-participation	NA
Randomization	In this large-scale observational study, we used a matching-based approach to disentangle contributions of income, education, grocery access, and fast food access on food consumption. To estimate the impact of each of these factors, we divide all available zip codes into treatment and control groups based on a median split; that is, we estimate the difference in outcomes between matched above-median and below-median zip codes. We create matched pairs of zip codes by selecting a zip code in the control group that is closely matched to the zip code in the treatment group across all factors, except the treatment factor of interest.

Reporting for specific materials, systems and methods

We require information from authors about some types of materials, experimental systems and methods used in many studies. Here, indicate whether each material, system or method listed is relevant to your study. If you are not sure if a list item applies to your research, read the appropriate section before selecting a response.

Materials & experimental systems

n/a	Involved in the study	
☒	☐	Antibodies
☒	☐	Eukaryotic cell lines
☒	☐	Palaeontology and archaeology
☒	☐	Animals and other organisms
☐	☒	Human research participants
☒	☐	Clinical data
☒	☐	Dual use research of concern

Methods

n/a	Involved in the study	
☒	☐	ChIP-seq
☒	☐	Flow cytometry
☒	☐	MRI-based neuroimaging

Human research participants

Policy information about studies involving human research participants

Population characteristics	Comparing our study population to nationally representative survey data, we found that our study population had significant overlap with the U.S. national population in terms of population demographics, education and weight status (Body Mass Index; BMI), but that it was skewed towards women and higher income (Supplementary Table e1). No individual-level demographics were collected and the sample was anonymized.
Recruitment	Participants were participants of the MyFitnessPal app, a free application for tracking caloric intake. We analyzed anonymized, retrospective data collected during a 7-year observation period between 2010 and 2016 that were aggregated to the zip code level.
Ethics oversight	Stanford University IRB

Note that full information on the approval of the study protocol must also be provided in the manuscript.
